# Author Correction: The ubiquitin ligase UBR5 suppresses proteostasis collapse in pluripotent stem cells from Huntington’s disease patients

**DOI:** 10.1038/s41467-020-14726-x

**Published:** 2020-02-17

**Authors:** Seda Koyuncu, Isabel Saez, Hyun Ju Lee, Ricardo Gutierrez-Garcia, Wojciech Pokrzywa, Azra Fatima, Thorsten Hoppe, David Vilchez

**Affiliations:** 1 Institute for Genetics and Cologne Excellence Cluster for Cellular Stress Responses in Aging-Associated Diseases (CECAD), University of Cologne, Joseph Stelzmann Strasse 26, 50931 Cologne, Germany; 2 Laboratory of Protein Metabolism in Development and Aging, International Institute of Molecular and Cell Biology, Trojdena Street 4, 02-109 Warsaw, Poland

**Keywords:** Ubiquitin ligases, Mechanisms of disease, Proteasome, Protein quality control, Huntington's disease

Correction to: *Nature Communications* 10.1038/s41467-018-05320-3, published online 23 July 2018.

The original version of this Article contained an error in Fig. 2e, in which the panel ‘Control #2 (Q33) treated with MG-132’ was also shown as ‘Control #2 (Q33) treated with DMSO’. This has been corrected in both the PDF and HTML versions of the Article.

