# Supplementary material for: The ubiquitin ligase UBR5 suppresses proteostasis collapse in pluripotent stem cells from Huntington’s disease patients
Source: Nat Commun. 2018 Jul 23;9:2886. doi: 10.1038/s41467-018-05320-3 (PMC6056416; doi:10.1038/s41467-018-05320-3)
Supplement: Supplementary file 1 — Supplementary Information [file 41467_2018_5320_MOESM1_ESM.pdf]

Supplementary Information

The ubiquitin ligase UBR5 suppresses proteostasis collapse in pluripotent stem cells from Huntington's disease patients

Koyuncu et al.

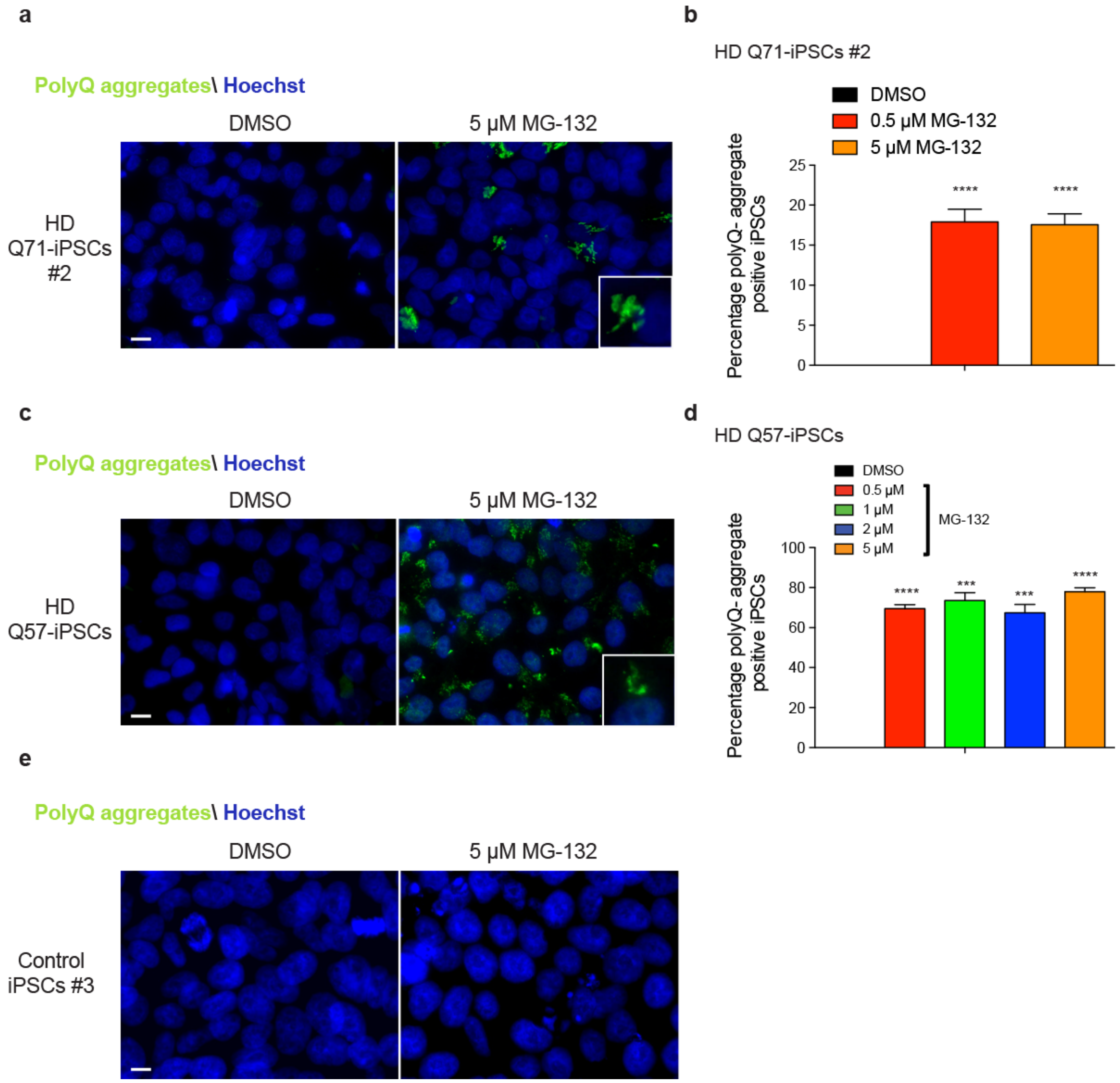

Supplementary Figure 1. Proteasome inhibition induces aggregation of polyQ-expanded HTT in distinct HD-iPSC lines. a, Immunocytochemistry with antibody against polyQ-expanded proteins of HD Q71-iPSC line #2 treated with 5 µM MG-132 for 12 h. Cell nuclei were stained with Hoechst 33342. The images are representative of four independent experiments. **b,** Graph represents the percentage of polyQ aggregate-positive cells/total nuclei in HD Q71-iPSC line #2 (mean ± s.e.m., 4 independent experiments, 300-350 total cells per treatment). **c,** Immunocytochemistry with antibody against polyQ-expanded proteins of HD Q57-iPSCs treated with 5 µM MG-132 (12 h). The images are representative of four independent experiments. **d,** Graph represents the percentage of polyQ aggregate-positive cells/total nuclei in HD Q57-iPSCs (mean ± s.e.m., 4 independent experiments, 250-300 total cells per treatment). **e,** Immunocytochemistry of control iPSC line #3 with antibody against polyQ-expanded proteins. The images are representative of two independent experiments. In **a**, **c** and **e**, scale bar represents 10 µm. All the statistical comparisons were made by Student's t-test for unpaired samples. P-value: *** (P<0.001), **** (P<0.0001).

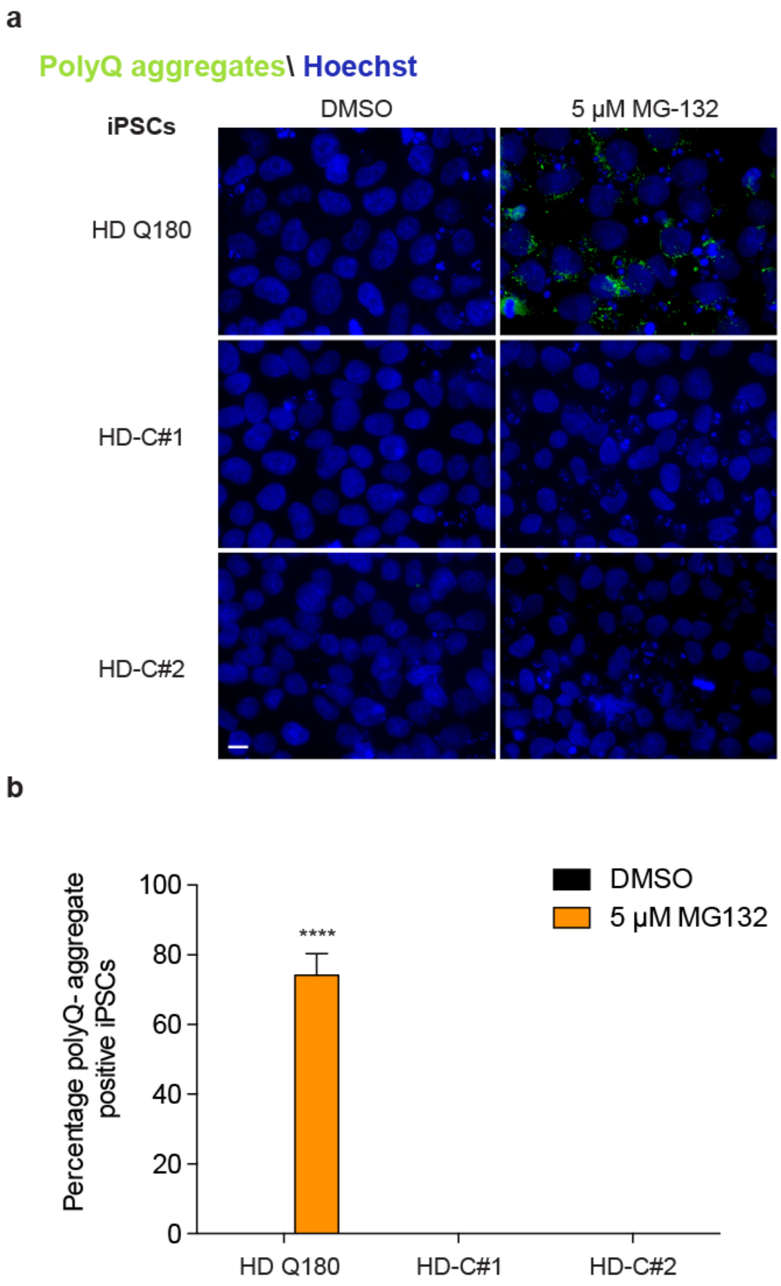

Supplementary Figure 2. Proteasome inhibition does not induce HTT aggregation in two corrected isogenic counterparts of Q180-iPSCs. a, Immunocytochemistry of Q180-iPSCs and two isogenic counterparts (*i.e.*, HD-C#1 and HD-C#2), in which the 180 CAG expansion was corrected to a nonpathological repeat length. Proteasome inhibition treatment: 5 μM MG-132 for 12 h. We used an antibody against polyQ-expanded protein to detect mutant HTT aggregates. Cell nuclei were stained with Hoechst 33342. Scale bar represents 10 μm. The images are representative of three independent experiments. **b,** Graph represents the percentage of polyQ aggregate-positive cells/total nuclei in Q180-iPSCs and their corrected isogenic counterparts (mean ± s.e.m., 3 independent experiments, 250-300 total cells per treatment for each line).

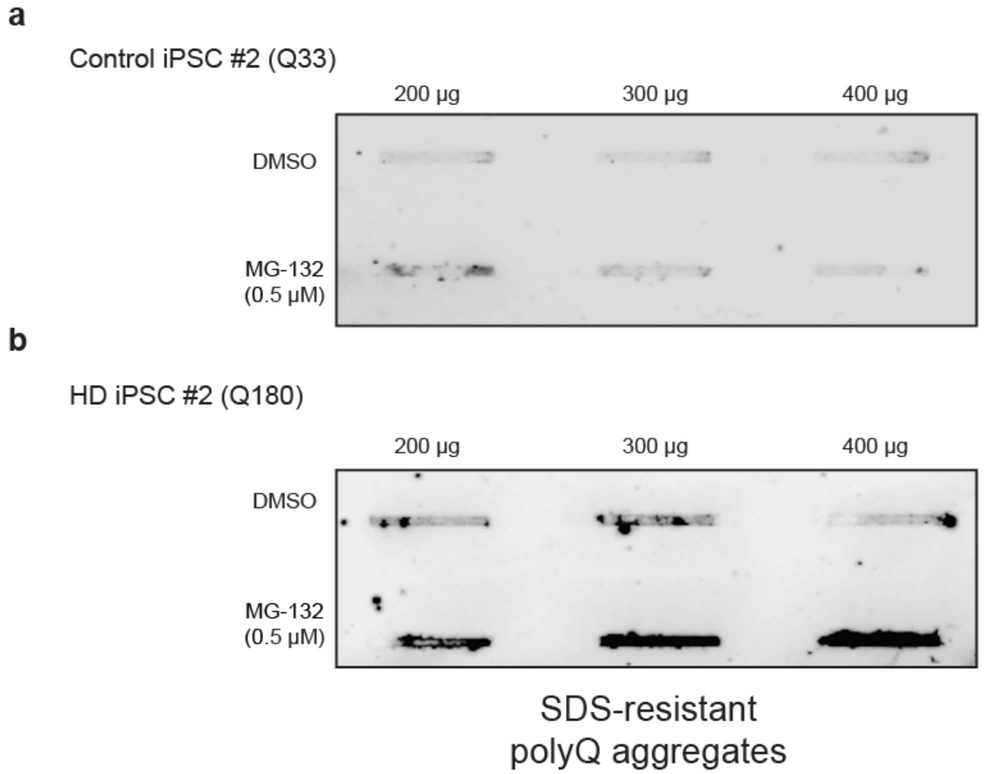

Supplementary Figure 3. Filter trap assay detects aggregation of endogenous polyQ-expanded HTT in HD iPSCs treated with proteasome inhibitor. a, Filter trap analysis of control iPSCs #2 (Q33) treated with MG-132 for 12 h using anti-polyQ-expansion diseases marker antibody. We did not observe differences when we loaded higher amounts of total protein from the cell lysate, indicating that the weak signal detected in this assay corresponds to background signal. **b,** Filter trap analysis of HD Q180-iPSCs treated with MG-132 for 12 h using anti-polyQ-expansion diseases marker. In MG-132-treated samples, the levels of detected polyQ-expanded aggregates correlate with the amount of total protein loaded. However, the background signal does not change in DMSO-treated cells regardless the total amount of protein loaded. The images are representative of two independent experiments.

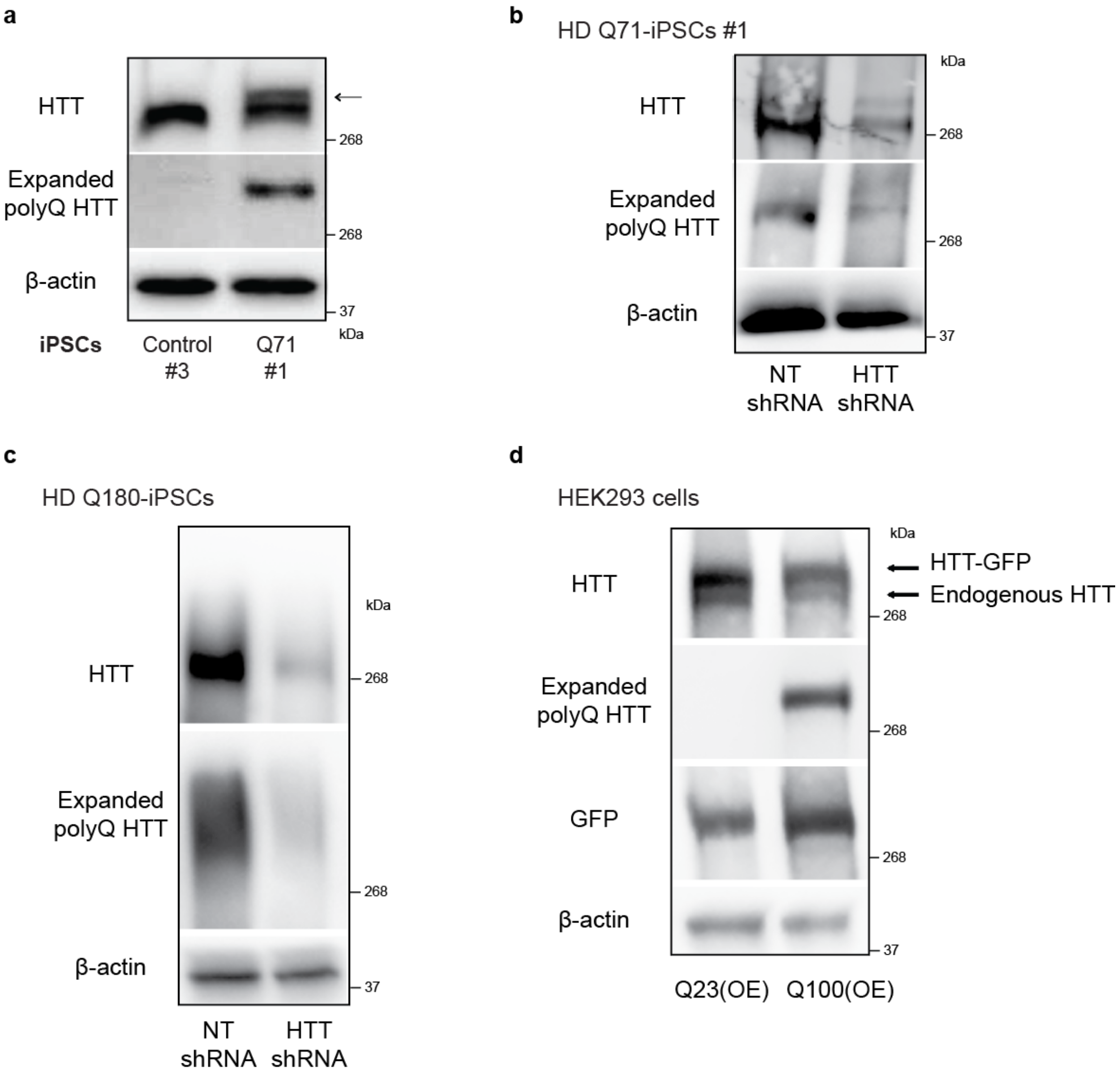

Supplementary Figure 4. Validation of antibodies to total HTT and anti-polyQ-expansion diseases marker. a, Western blot analysis of control iPSCs #3 and HD Q71-iPSCs #1 with antibodies to total HTT, polyQ-expanded proteins and β-actin. Arrow indicates mutant HTT detected with total HTT antibody in the HD Q71-iPSC line. The images are representative of three independent experiments. **b,** Western blot analysis of HD Q71-iPSCs #1 upon HTT knockdown. The images are representative of two independent experiments. **c,** Western blot analysis of HD Q180-iPSCs upon HTT knockdown. The images are representative of two independent experiments. **d,** Western blot analysis of Q23-HTT and Q100-HTT overexpressing (OE)-HEK293 cells with antibodies to total HTT, polyQ-expanded proteins, GFP and β-actin. The images are representative of two independent experiments.

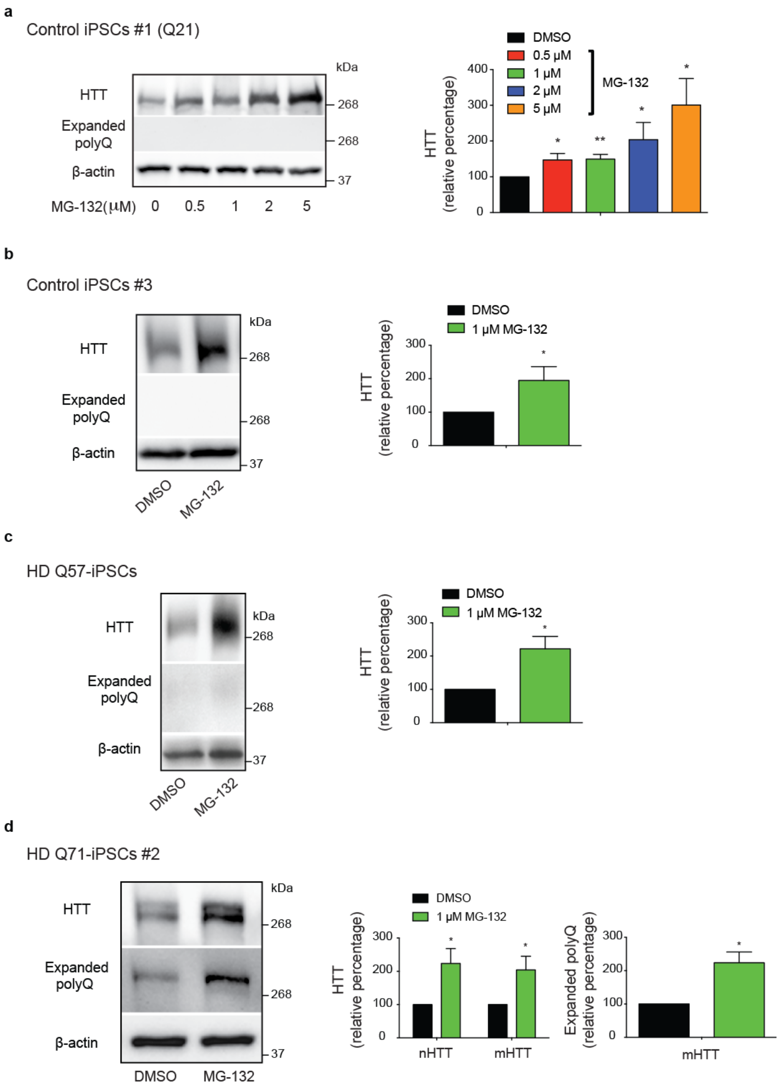

Supplementary Figure 5. Proteasome inhibition increases HTT protein levels in distinct iPSC lines. a, Western blot analysis with antibodies to total HTT and polyQ-expanded proteins of control iPSC line #1 treated with different concentrations of MG-132 (12 h). The graph represents the HTT relative percentage values to DMSO-treated iPSCs corrected for β-actin loading control (mean ± s.e.m. of three independent experiments). **b,** Western blot analysis of control iPSC line #3 treated with 1 μM MG-132 (12 h). The graph represents the HTT relative percentage values (corrected for β-actin) to DMSO-treated iPSCs (mean ± s.e.m. of three independent experiments). **c,** Western blot analysis of HD Q57-iPSC line treated with 1 μM MG-132 for 12 h. The graph represents the relative percentage value to DMSO-treated iPSCs (corrected for β-actin) of total HTT levels detected with anti-HTT antibody (mean ± s.e.m. of three independent experiments). **d,** Western blot analysis of HD Q71-iPSC line #2 treated with 1 μM MG-132 for 12 h. The graphs represent the relative percentage values to DMSO-treated iPSCs (corrected for β-actin) of normal (nHTT) and mutant HTT (mHTT) detected with antibodies to total HTT and polyQ-expanded proteins (mean ± s.e.m. of three independent experiments). All the statistical comparisons were made by Student's t-test for unpaired samples. P-value: *(P<0.05), **(P<0.05).

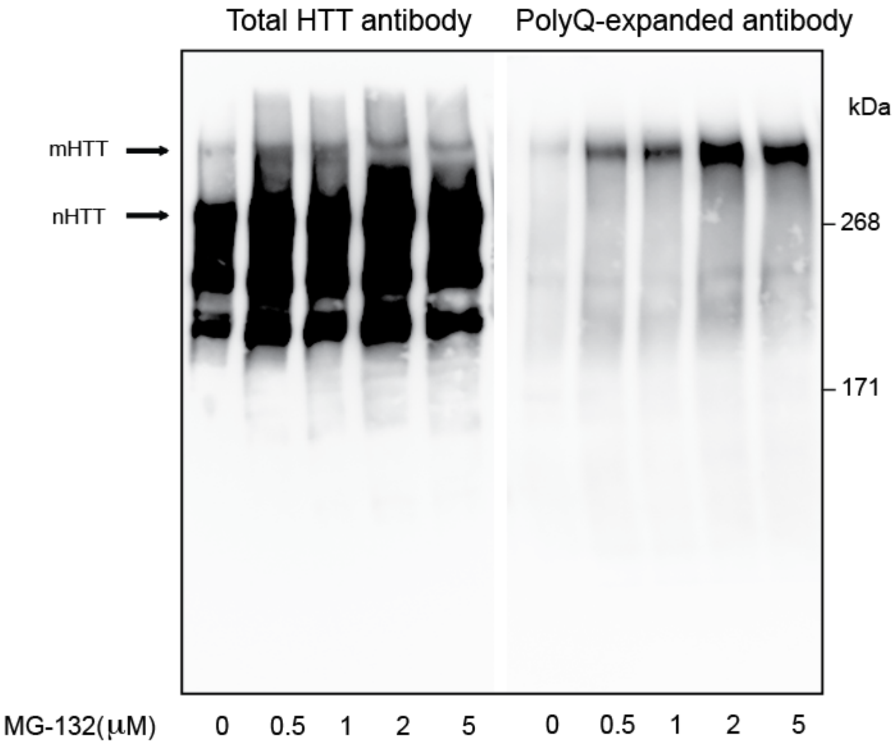

Supplementary Figure 6. Higher exposure time of the membrane presented in **Fig. 2e** for a better comparison of mutant HTT (mHTT) levels detected with total HTT antibody in HD Q180-iPSCs.

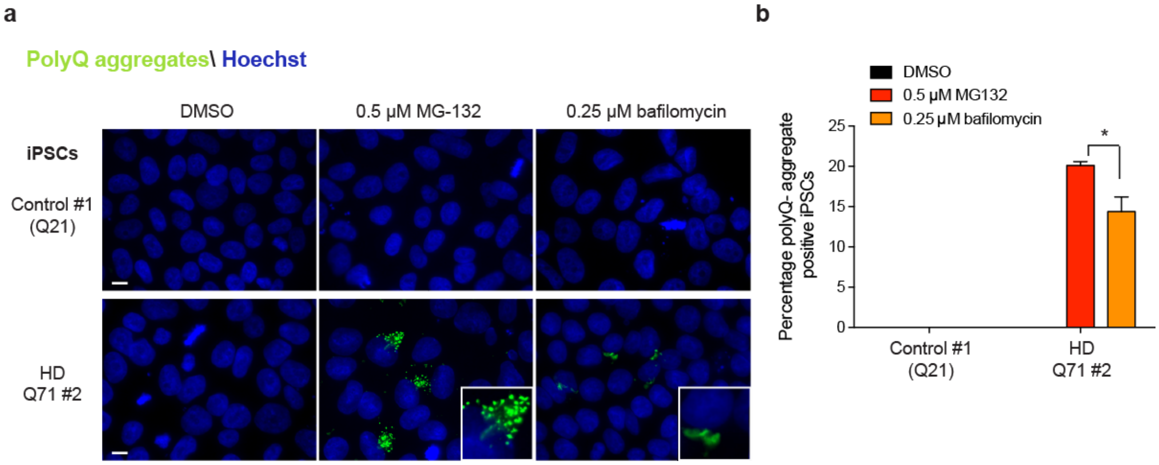

Supplementary Figure 7. Autophagy inhibition triggers accumulation of polyQ-expanded HTT aggregates to a lesser extent when compared to proteasome inhibition. a, Immunocytochemistry of control iPSC line #1 (Q21) and HD Q71-iPSC line #2 treated with 0.5 µM MG-132 or 0.25 µM bafilomycin for 12 h. We used an antibody against polyQ-expanded protein to detect mutant HTT aggregates. Cell nuclei were stained with Hoechst 33342. Scale bar represents 10 µm. The images are representative of three independent experiments. **b,** Graph represents the percentage of polyQ aggregate-positive cells/total nuclei in the indicated iPSC lines with 0.5 µM MG-132 or 0.25 µM bafilomycin for 12 h (mean ± s.e.m., 3 independent experiments, 200-250 total cells per treatment for each line).

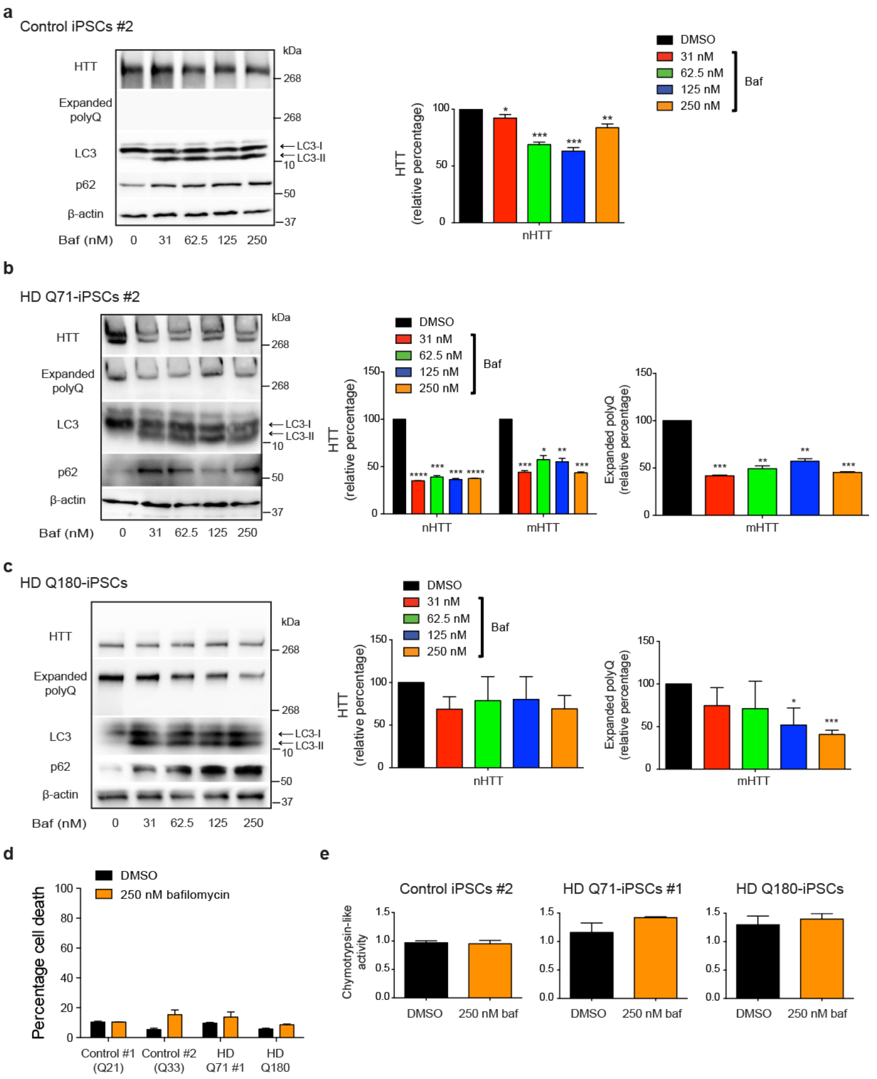

Supplementary Figure 8. Autophagy inhibition does not increase HTT protein levels in iPSCs. a, Western blot analysis of control iPSC line #2 treated with bafilomycin (12 h) using antibodies to total HTT, polyQ-expanded proteins, LC3 and P62. β-actin is the loading control. The graph represents the relative percentage values to DMSO-treated iPSCs of nHTT detected with total HTT antibody and corrected for β-actin loading control (mean ± s.e.m. of three independent experiments). **b,** Western blot analysis of HD Q71-iPSC line #2 treated with bafilomycin (12 h) The graphs represent the relative percentage values to DMSO-treated iPSCs (corrected for β-actin) of normal (nHTT) and mutant (mHTT) huntingtin detected with antibodies to total HTT and polyQ-expanded proteins (mean ± s.e.m. of three independent experiments). **c,** Western blot analysis of HD Q180-iPSCs treated with bafilomycin (12 h). The graphs represent the relative percentage values to DMSO-treated iPSCs (corrected for β-actin) of nHTT and mHTT detected with antibodies to total HTT and polyQ-expanded proteins, respectively (mean ± s.e.m. of three independent experiments). **d,** Graph represents the percentage of propidium iodide-positive cells/total nuclei in the indicated iPSC lines treated with bafilomycin (12 h) (mean ± s.e.m., 3 independent experiments, 200-300 total cells for each line). **e,** Chymotrypsin-like proteasome activity in the indicated iPSC lines treated with bafilomycin for 12 h (relative slope to the respective DMSO-iPSCs). Graph represents the mean ± s.e.m. of three independent experiments. All the statistical comparisons were made by Student's t-test for unpaired samples. P-value: *(P<0.05), **(P<0.01), ***(P<0.001), **** (P<0.0001).

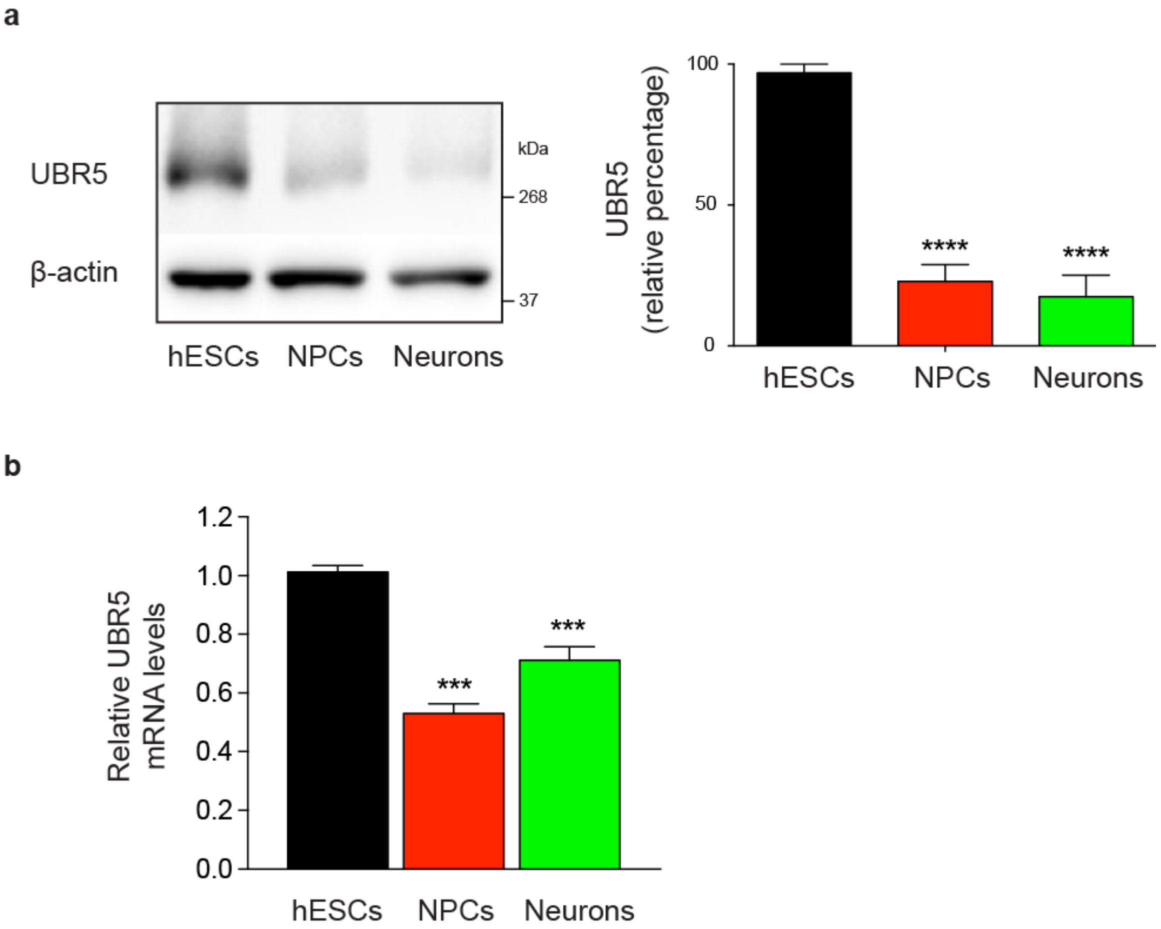

Supplementary Figure 9. UBR5 levels decrease during differentiation of H9 hESCs. a, Western blot analysis of UBR5 levels in H9 hESCs compared with their neural progenitor cell (NPC) and terminally differentiated neuronal counterparts. β-actin is the loading control. The graph represents the UBR5 relative percentage values (corrected for β-actin loading control) to H9 hESCs (mean ± s.e.m. of five independent experiments). **b,** qPCR of UBR5 mRNA levels. Graph represents the mean ± s.e.m. (relative expression to H9 hESCs) of three independent experiments. All the statistical comparisons were made by Student's t-test for unpaired samples. P-value: ***(P<0.001), ****(P<0.0001).

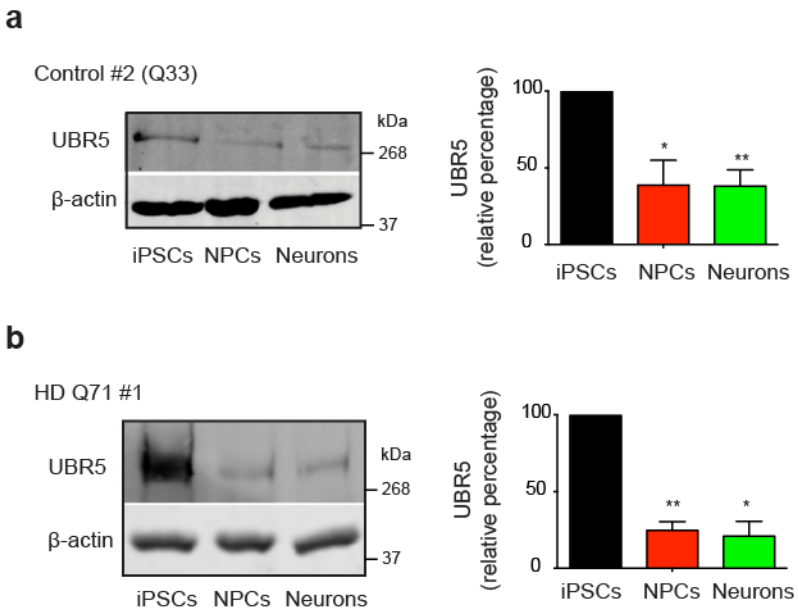

Supplementary Figure 10. UBR5 levels decrease during differentiation of control and HD-iPSC lines. a, Western blot of UBR5 levels in control #2 iPSCs (Q33) compared with their neural progenitor cell (NPC) and striatal neuron counterparts. The graph represents the UBR5 relative percentage values to iPSCs corrected for β-actin loading control (mean ± s.e.m. of three independent experiments). **b,** Western blot of UBR5 levels in HD Q71 iPSCs #1 compared with their NPC and striatal neuronal counterparts. The graph represents the UBR5 relative percentage values to iPSCs corrected for β-actin loading control (mean ± s.e.m. of three independent experiments). All the statistical comparisons were made by Student's t-test for unpaired samples. P-value: *(P<0.05), ** (P<0.01).

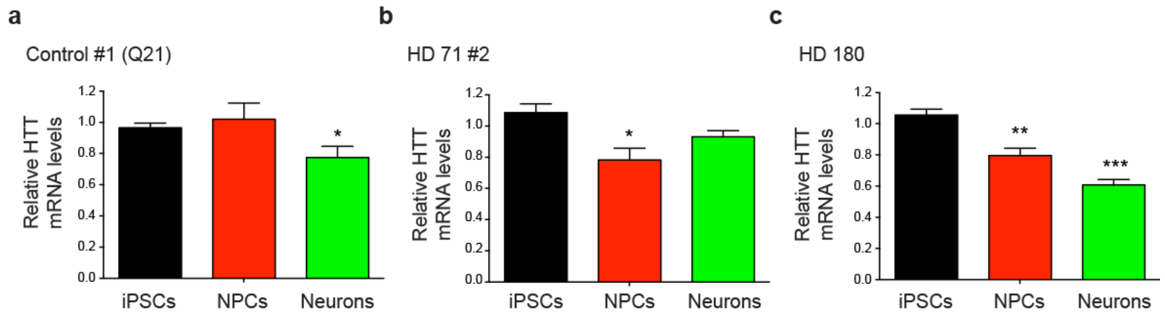

Supplementary Figure 11. Analysis of HTT mRNA levels in iPSCs and their differentiated counterparts. qPCR of HTT mRNA levels in (**a**) control #1 (Q21), (**b**) HD 71 #2 and (**c**) HD 180. Graphs represents the mean ± s.e.m. (relative expression to iPSCs) of three independent experiments. All the statistical comparisons were made by Student's t-test for unpaired samples. P-value: *(P<0.05), **(P<0.01), ***(P<0.001).

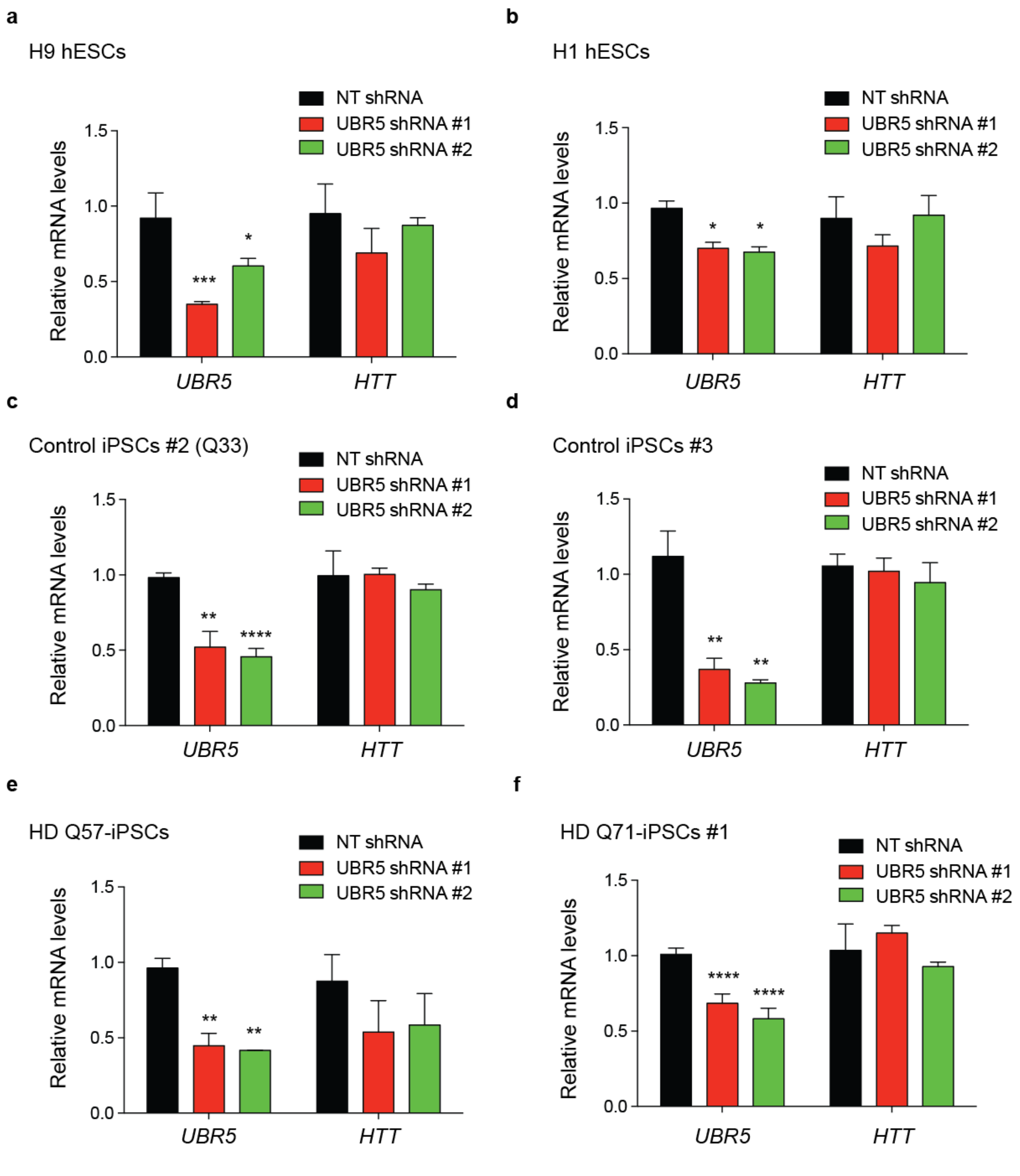

Supplementary Figure 12. Loss of intrinsic high levels of UBR5 does not change HTT mRNA levels in human pluripotent stem cells. a, qPCR analysis of UBR5 and HTT mRNA levels in H9 hESCs. Graph (relative expression to non-targeting (NT) shRNA) represents the mean ± s.e.m. of four independent experiments. **b,** qPCR analysis in H1 hESCs (mean ± s.e.m, n= 3 independent experiments). **c,** qPCR analysis in control iPSCs #2 (mean ± s.e.m, n= 4 independent experiments). **d,** qPCR analysis in control iPSCs #3 (mean ± s.e.m, n= 3 independent experiments). **e,** qPCR analysis in HD Q57-iPSCs (mean ± s.e.m, n= 3 independent experiments). **f,** qPCR analysis in HD Q71-iPSCs #1 (mean ± s.e.m, n= 5 independent experiments). All the statistical comparisons were made by Student's t-test for unpaired samples. P-value: *(P<0.05), **(P<0.01), ***(P<0.001), **** (P<0.0001).

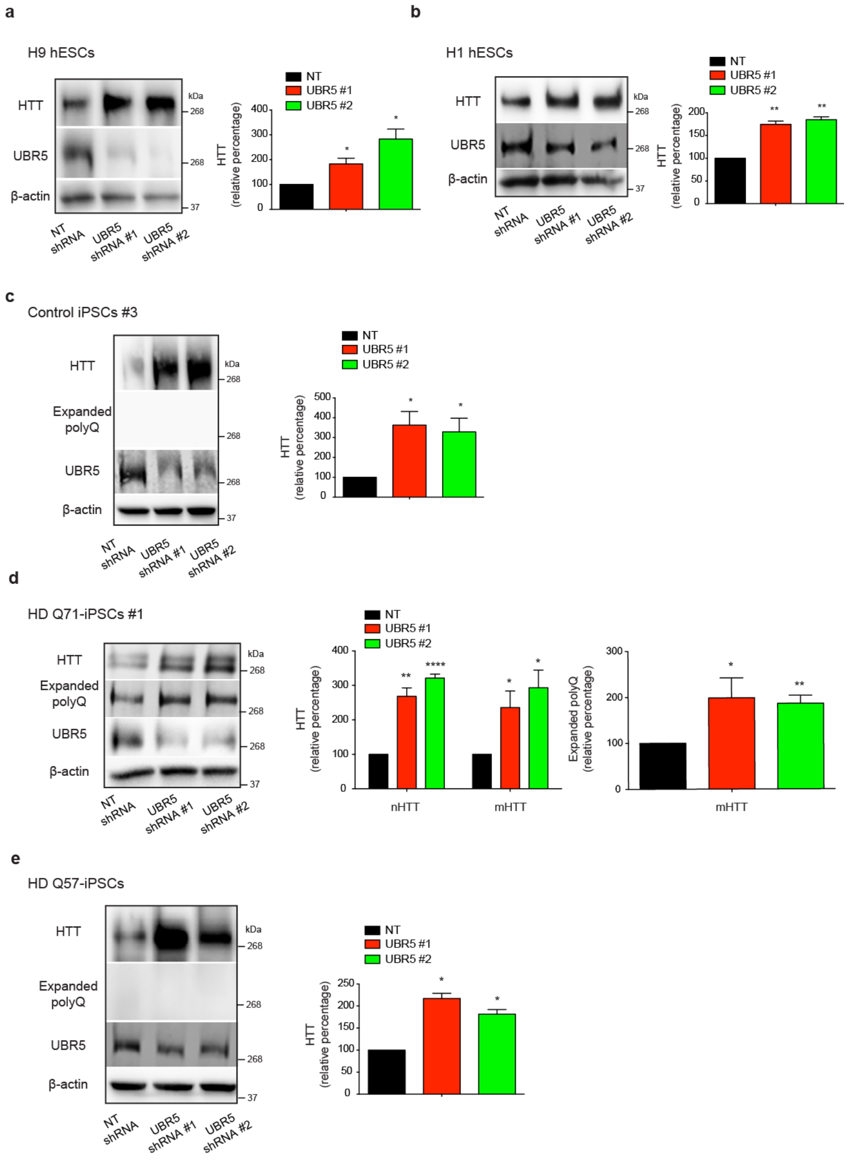

Supplementary Figure 13. Knockdown of UBR5 results in increased levels of HTT in hESCs, control iPSCs and HD iPSCs. a, Western blot analysis of HTT levels in H9 hESCs upon UBR5 knockdown. The graph represents the HTT relative percentage values to NT shRNA-H9 hESCs corrected for β-actin loading control (mean ± s.e.m. of three independent experiments). **b,** Western blot analysis of H1 hESCs. The graph represents the HTT relative percentage values to NT shRNA-H1 hESCs corrected for β-actin loading control (mean ± s.e.m. of two independent experiments). **c,** Western blot analysis of control iPSCs #3. The graph represents the HTT relative percentage values to NT shRNA-control iPSCs #3 corrected for β-actin loading control (mean ± s.e.m. of two independent experiments). **d,** Western blot analysis of HD Q71-iPSC line #1 upon UBR5 knockdown. The graphs represent the relative percentage values to NT shRNA-iPSCs (corrected for β-actin) of nHTT and mHTT detected with antibodies to total HTT and polyQ-expanded proteins (mean ± s.e.m. of three independent experiments). **e,** Western blot analysis of HD Q57-iPSCs. The graph represents the relative percentage value to NT shRNA-iPSCs (corrected for β-actin) of total HTT levels detected with anti-HTT antibody (mean ± s.e.m. of two independent experiments). All the statistical comparisons were made by Student's t-test for unpaired samples. P-value: * ($P<0.05$), **($P<0.01$), **** ($P<0.0001$).

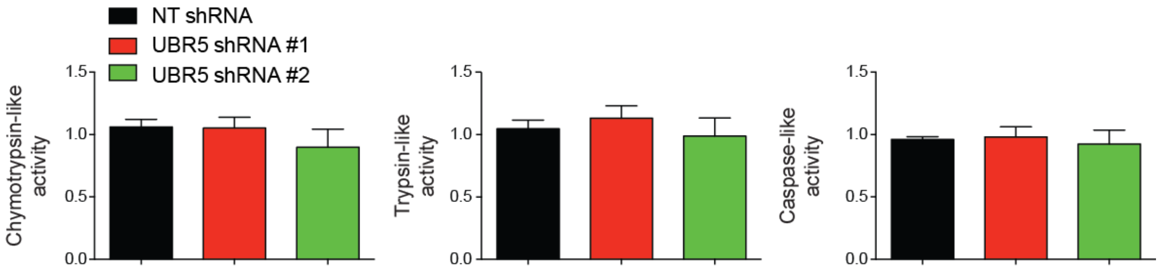

Supplementary Figure 14. Knockdown of UBR5 does not change proteasome activities in iPSCs. Proteasome activities in control iPSC line #2 (Q33) upon UBR5 knockdown (relative slope to NT shRNA iPSCs). Graphs represent the mean ± s.e.m. of three independent experiments. All the statistical comparisons were made by Student's t-test for unpaired samples.

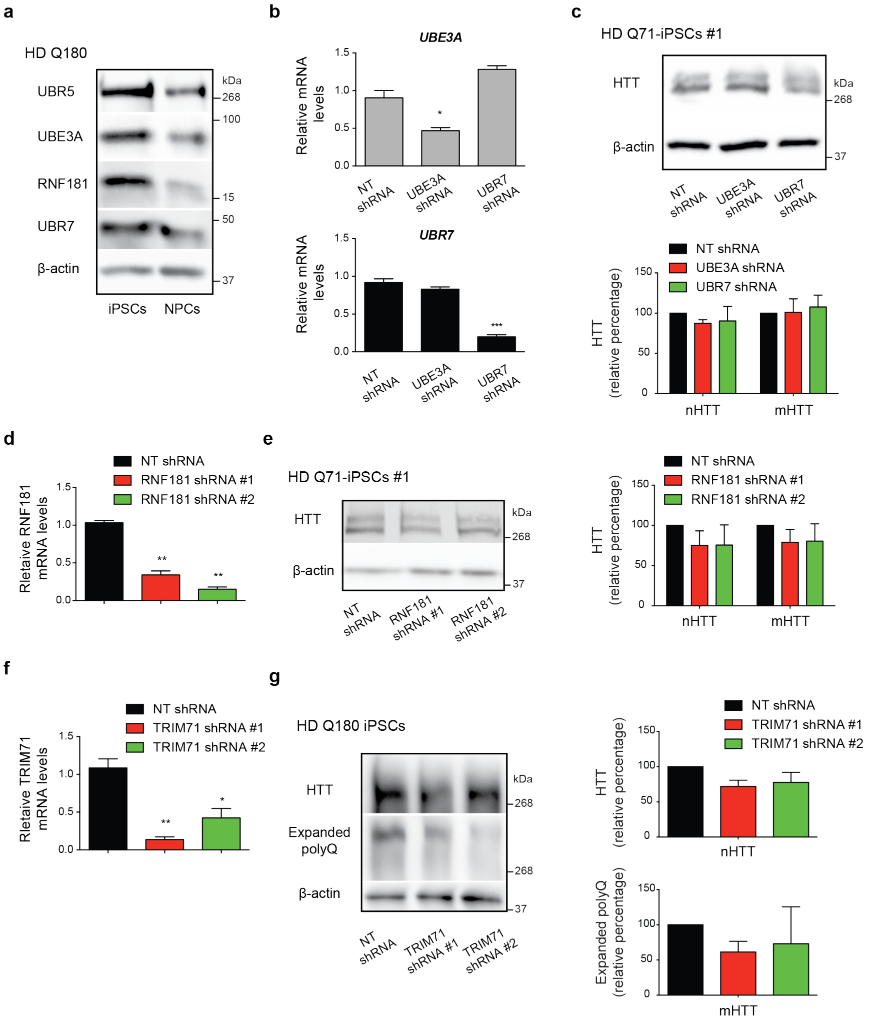

Supplementary Figure 15. Besides UBR5, knockdown of other up-regulated E3 enzymes in iPSCs does not increase HTT protein levels. a, Western blot analysis of HD Q180-iPSC lysates with antibodies to UBR5, UBE3A, RNF181 and UBR7 E3 enzymes. β-actin is the loading control. The images are representative of two independent experiments. **b,** qPCR analysis of UBE3A and UBR7 mRNA levels in HD Q71-iPSC line #3. Graph (relative expression to non-targeting (NT) shRNA) represents the mean ± s.e.m. of three independent experiments. **c,** Western blot analysis of HD Q71-iPSC line #1 upon knockdown of either UBE3A or UBR7. The graph represents the relative percentage values to NT shRNA iPSCs (corrected for β-actin) of nHTT and mHTT detected with antibody to total HTT (mean ± s.e.m. of two independent experiments). **d,** qPCR analysis of RNF181 mRNA levels in HD Q71-iPSC line #1. Graph (relative expression to NT shRNA) represents the mean ± s.e.m. of two independent experiments. **e,** Western blot analysis of HTT levels in HD Q71-iPSC line #1 upon knockdown of RNF181. The graph represents the relative percentage values to NT shRNA iPSCs (corrected for β-actin) of nHTT and mHTT detected with antibody to total HTT (mean ± s.e.m. of two independent experiments). **f,** qPCR analysis of TRIM71 mRNA levels in HD Q180-iPSCs. Graph (relative expression to NT shRNA) represents the mean ± s.e.m. of two independent experiments. **g,** Western blot analysis of HD Q180-iPSCs upon knockdown of TRIM71 with antibodies to total HTT, polyQ-expanded proteins and β-actin. The graphs represent the relative percentage values to NT shRNA iPSCs (corrected for β-actin) of nHTT and mHTT detected with antibodies to total HTT and polyQ-expanded proteins, respectively (mean ± s.e.m. of three independent experiments). All the statistical comparisons were made by Student's t-test for unpaired samples. P-value: *(P<0.05), **(P<0.01), ***(P<0.001).

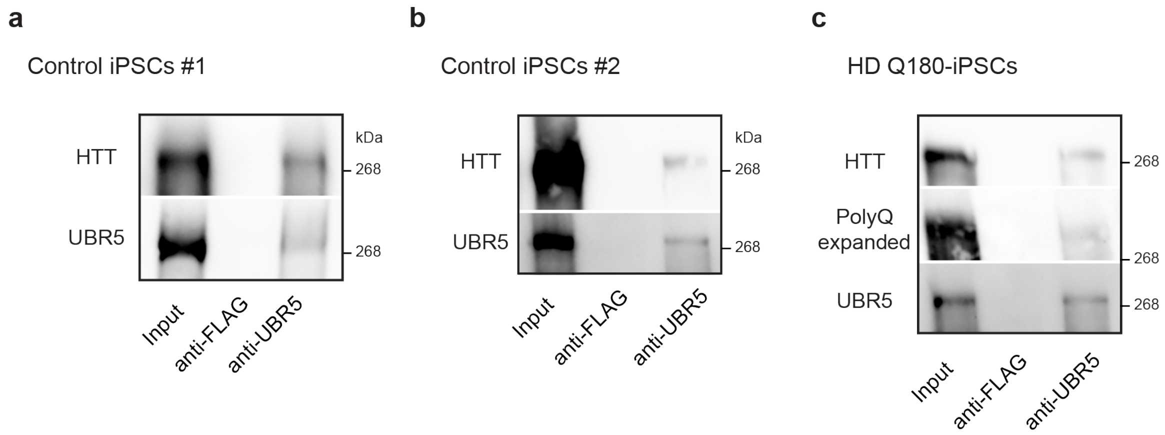

Supplementary Figure 16. UBR5 binds HTT protein in iPSCs. a, Co-immunoprecipitation with UBR5 and FLAG antibodies in control iPSC line #1 (Q21) followed by western blot with HTT and UBR5 antibodies. The images are representative of two independent experiments. **b,** Co-immunoprecipitation with UBR5 and FLAG antibodies in control iPSC line #2 (Q33). The images are representative of two independent experiments. **c,** Co-immunoprecipitation with UBR5 and FLAG antibodies in HD Q180-iPSCs followed by western blot with HTT, polyQ-expanded and UBR5 antibodies. The images are representative of two independent experiments.

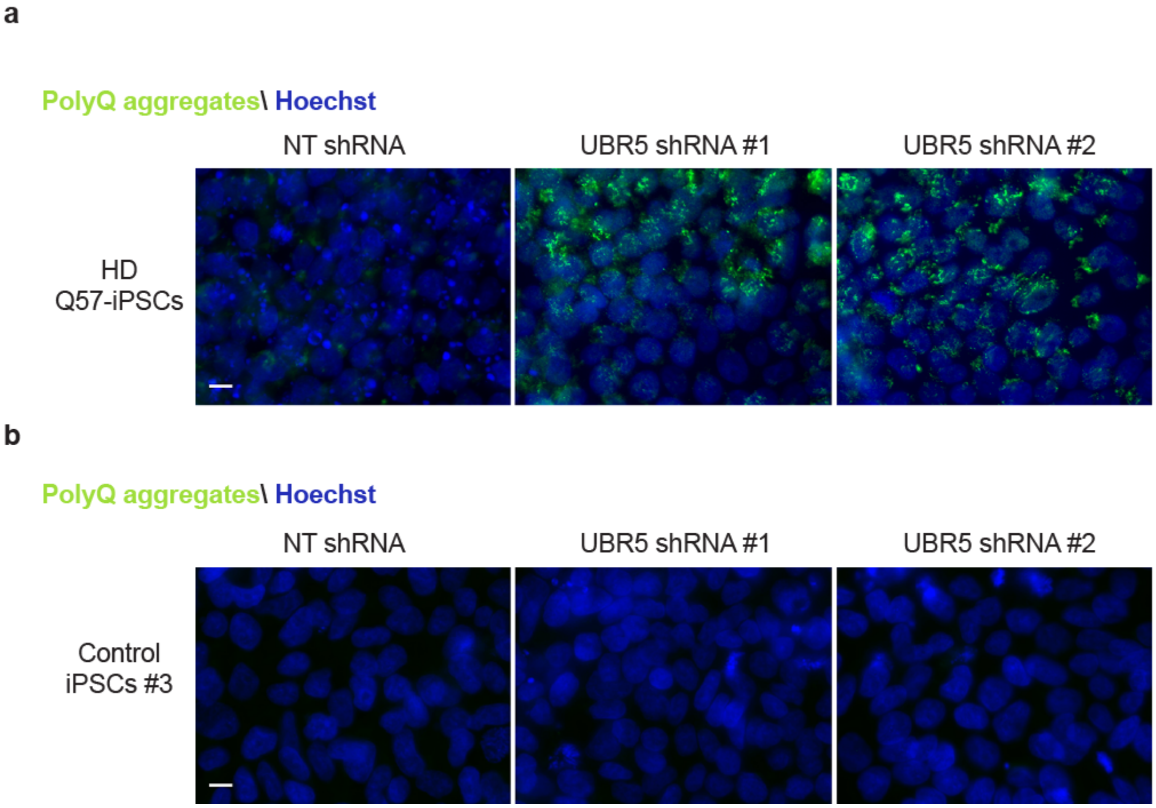

Supplementary Figure 17. UBR5 prevents accumulation of mutant HTT aggregates in HD-iPSCs. a, Immunocytochemistry of HD Q57-iPSCs upon UBR5 knockdown. PolyQ-expanded and Hoechst 33342 staining were used as markers of aggregates and nuclei, respectively. Scale bar represents 10 µm. The images are representative of three independent experiments. **b,** Immunocytochemistry of control iPSC line #3 upon UBR5 knockdown. Scale bar represents 10 µm. The images are representative of two independent experiments.

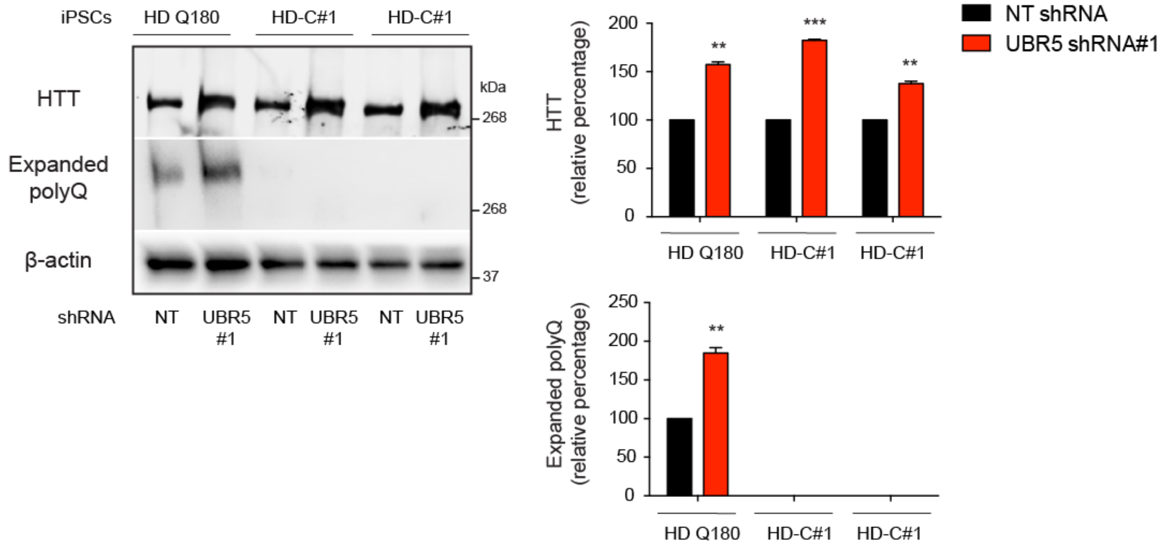

Supplementary Figure 18. Knockdown of UBR5 induces accumulation of normal HTT in corrected isogenic counterparts of Q180-iPSCs. Western blot of Q180-iPSCs and two isogenic counterparts (*i.e.*, HD-C#1 and HD-C#2), in which the 180 CAG expansion was corrected to a nonpathological repeat length. The graphs represent the relative percentage values to the respective NT shRNA-iPSCs (corrected for β-actin) of nHTT and mHTT detected with antibodies to total HTT and polyQ-expanded proteins, respectively (mean ± s.e.m. of three independent experiments).

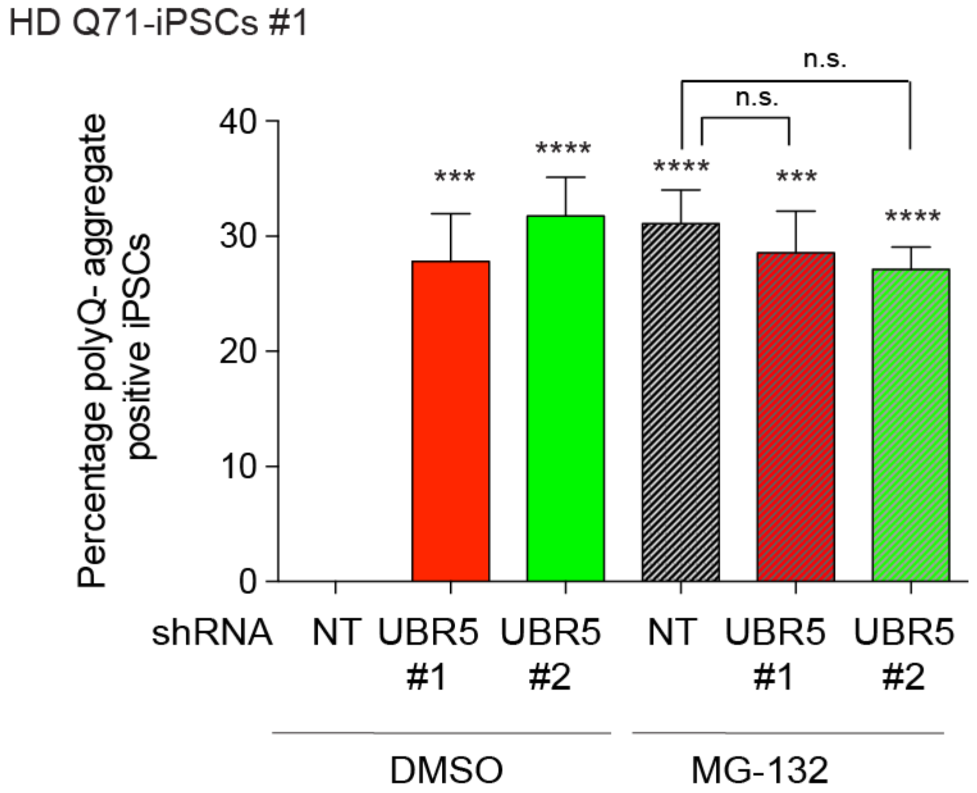

Supplementary Figure 19. Proteasome inhibition does not further increase the accumulation of polyQ-aggregates induced by loss of UBR5. Graph represents the percentage of polyQ aggregate-positive cells/total nuclei in HD Q71-iPSC line #1 (mean ± s.e.m., 3 independent experiments, 300-400 total cells per condition). Proteasome inhibitor treatment: 5 μM MG-132 for 12 h. PolyQ-expanded and Hoechst 33342 staining were used as markers of aggregates and nuclei, respectively. All the statistical comparisons were made by Student's t-test for unpaired samples. P-value: *** (P<0.001), **** (P<0.0001).

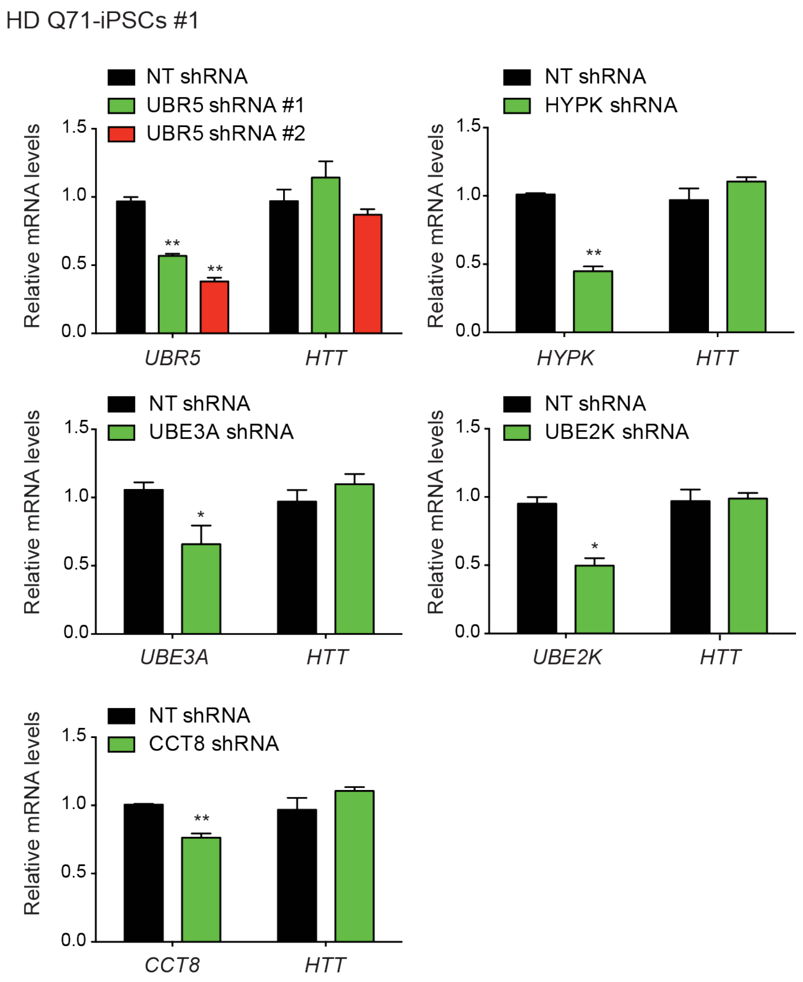

Supplementary Figure 20. Knockdown of distinct proteostasis components in HD-iPSCs does not impair HTT mRNA levels. qPCR analysis of HD Q71-iPSC line #1 upon knockdown of distinct proteostasis components. Graph (relative expression to non-targeting (NT) shRNA) represents the mean ± s.e.m. of three independent experiments. All the statistical comparisons were made by Student's t-test for unpaired samples. P-value: * ($P<0.05$), ** ($P<0.01$).

a

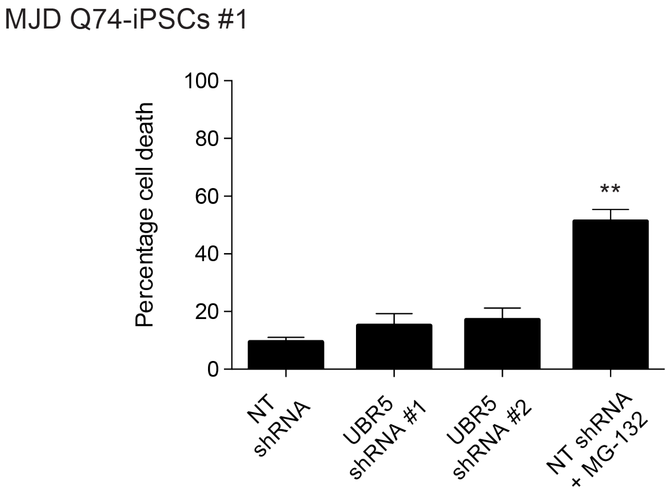

b

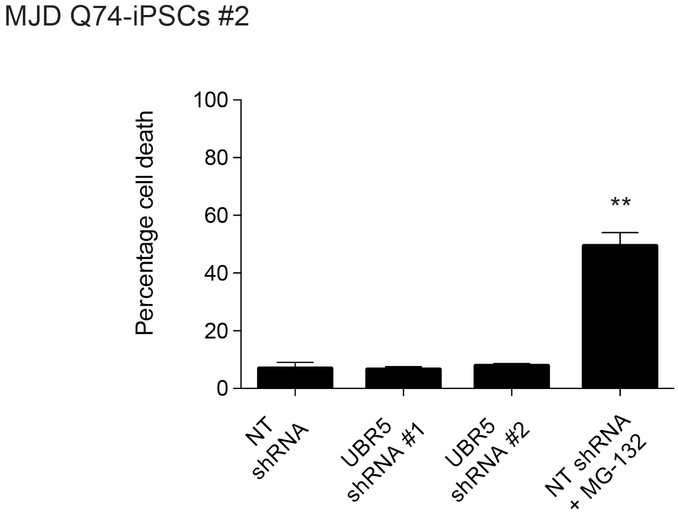

Supplementary Figure 21. Percentage of cell death in MJD-iPSC lines. a, Graph represents the percentage of propidium iodide-positive cells/total nuclei in MJD-iPSC line #1 (mean ± s.e.m., 2 independent experiments, 400-500 total cells). **b,** Percentage of propidium iodide-positive cells/total nuclei in MJD-iPSC line #2 (mean ± s.e.m., 2 independent experiments, 400-500 total cells). Proteasome inhibitor treatment: 5 µM MG-132 for 6 h. All the statistical comparisons were made by Student's t-test for unpaired samples. P-value: ** ($P<0.01$).

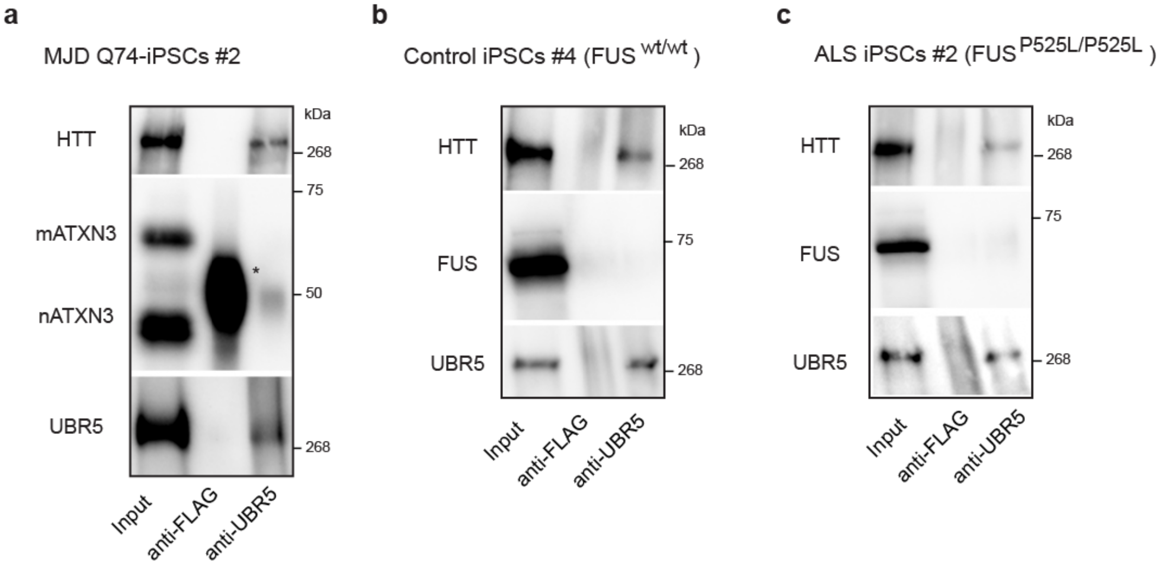

Supplementary Figure 22. UBR5 does not interact with ATXN3 and FUS proteins in iPSCs. **a,** Co-immunoprecipitation with UBR5 and FLAG antibodies in MJD Q74-iPSC line #2 followed by western blot with HTT, ATXN3 and UBR5 antibodies. * indicates IgG. The images are representative of two independent experiments. **b,** Co-immunoprecipitation with UBR5 and FLAG antibodies in control iPSCs #4 ($FUS^{wt/wt}$), followed by western blot with HTT, FUS and UBR5 antibodies. The images are representative of two independent experiments. **c,** Co-immunoprecipitation with UBR5 and FLAG antibodies in ALS-iPSCs #2 ($FUS^{P525L/P525L}$), followed by western blot with HTT, FUS and UBR5 antibodies. The images are representative of two independent experiments.

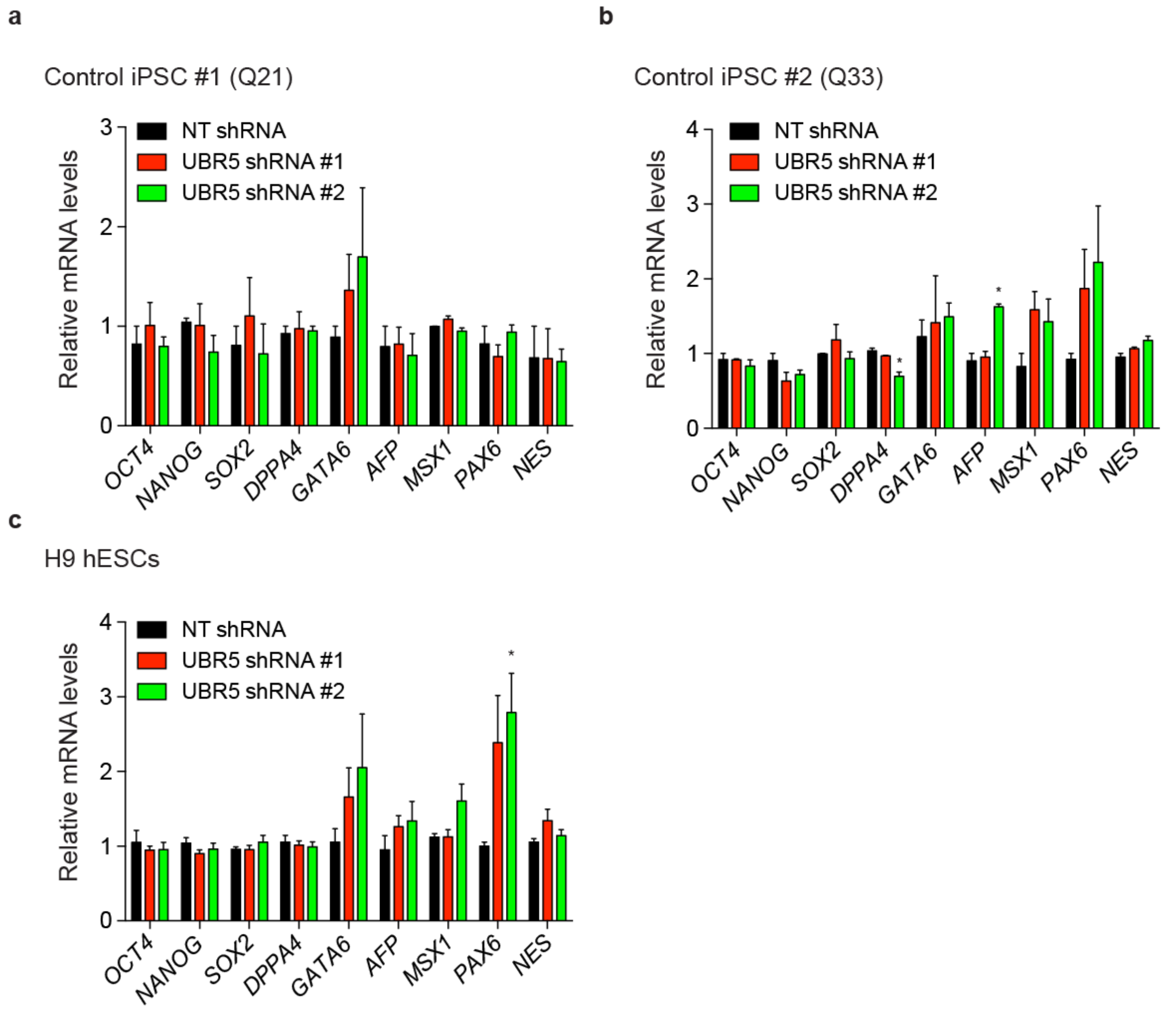

Supplementary Figure 23. Loss of UBR5 does not affect expression of pluripotency and germ layer markers in control pluripotent stem cells. a, qPCR analysis in control iPSC line #1 (Q21). The graph represents the relative expression to NT shRNA (mean ± s.e.m., n= 3 independent experiments). **b,** Control iPSC line #2 (Q33) (mean ± s.e.m., n= 3 independent experiments). **c,** H9 hESCs (mean ± s.e.m., n= 4 independent experiments). All the statistical comparisons were made by Student's t-test for unpaired samples. P-value: * ($P<0.05$).

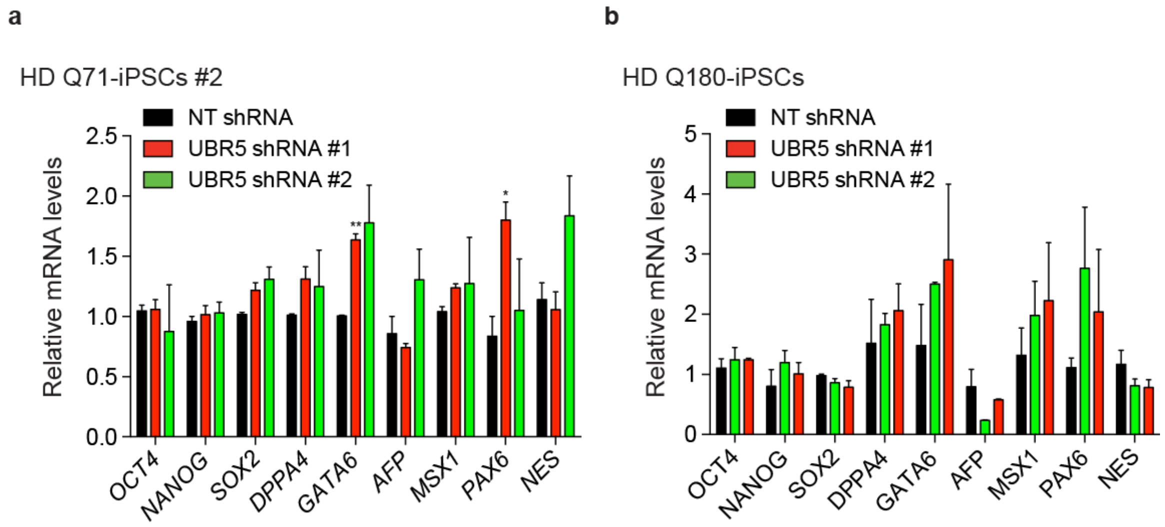

Supplementary Figure 24. Accumulation of polyQ-expanded HTT aggregates upon UBR5 knockdown does not affect pluripotency expression levels. qPCR analysis of pluripotency markers in (**a**) HD Q71-iPSC line #2 and (**b**) HD Q180-iPSC line. The graphs represent the relative expression to NT shRNA HD-iPSCs (mean ± s.e.m. (n= 3 independent experiments for each line)). All the statistical comparisons were made by Student's t-test for unpaired samples. P-value: * (P<0.05), ** (P<0.01).

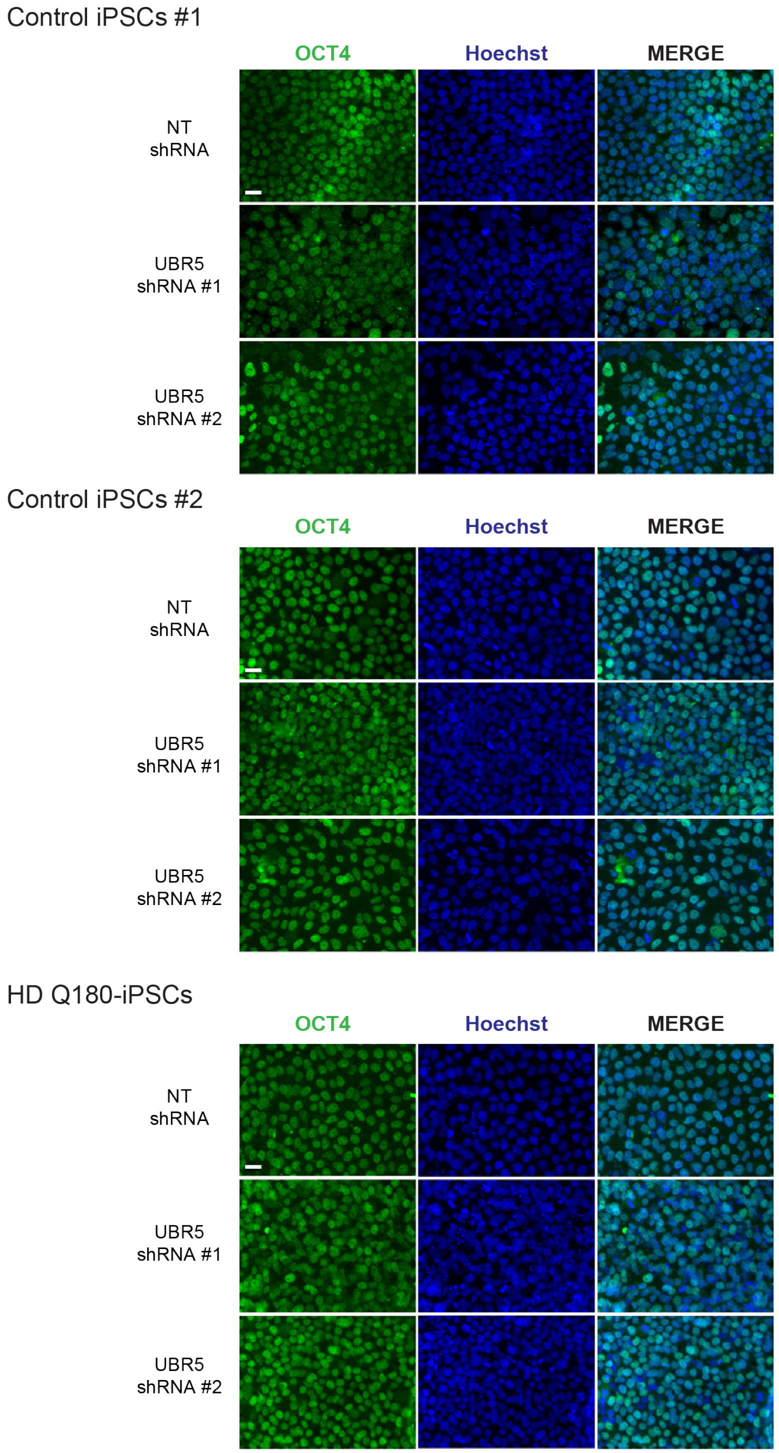

Supplementary Figure 25. UBR5 knockdown does not induce loss of OCT4 expression. Immunocytochemistry of control iPSCs #1, control iPSCs #2 and HD Q180-iPSCs. OCT4 and Hoechst 33342 staining were used as markers of pluripotency and nuclei, respectively. Scale bar represents 20 µm. The images are representative of three independent experiments.

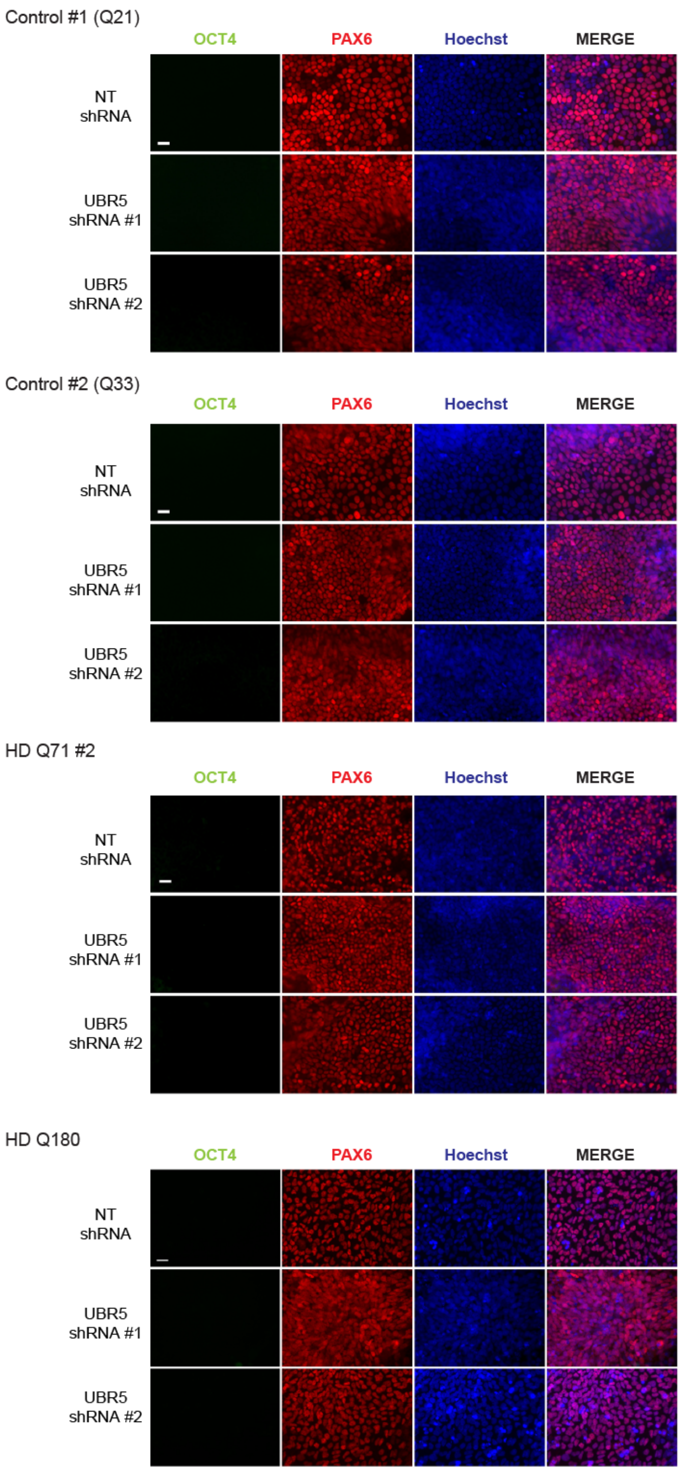

Supplementary Figure 26. Loss of UBR5 does not impair neural differentiation of control and HD-iPSCs. After neural induction of the indicated iPSCs lines with down-regulated levels of UBR5, we did not observe differences in their ability to differentiate into PAX6-positive cells compared with their respective NT shRNA controls. Scale bar represents 20 µm. The images are representative of three independent experiments.

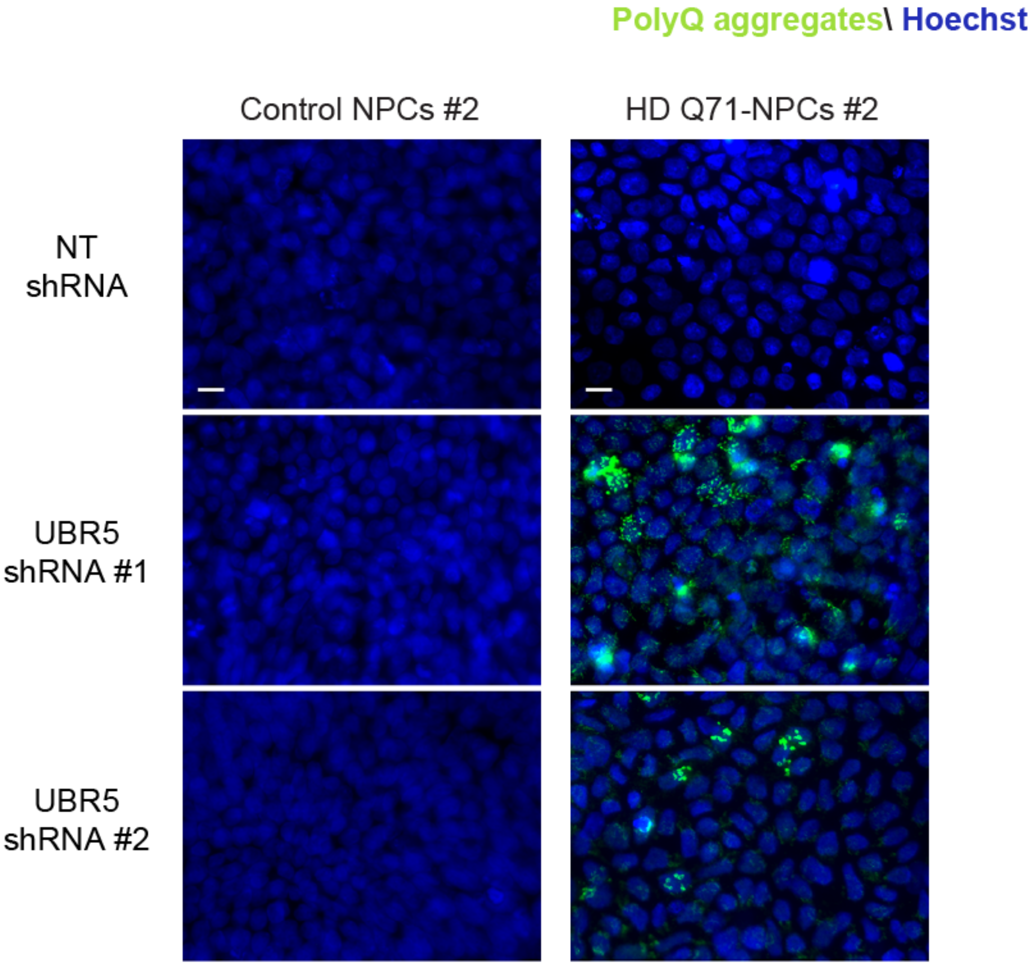

Supplementary Figure 27. HD-iPSCs with downregulated levels of UBR5 differentiate into NPCs with aggregates. Upon neural induction of HD Q71-iPSC line #2 with down-regulated levels of UBR5, NPCs accumulate polyQ-expanded HTT aggregates. PolyQ-expanded and Hoechst 33342 staining were used as markers of aggregates and nuclei, respectively. Scale bar represents 10 µm. The images are representative of three independent experiments.

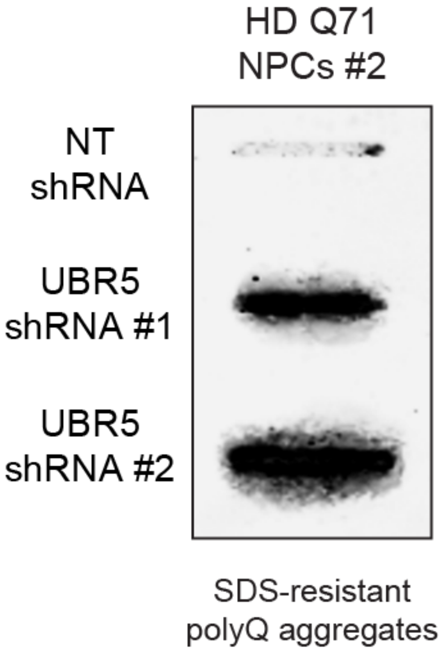

Supplementary Figure 28. Filter trap of HD Q71-NPCs #2 derived from iPSCs with downregulated levels of UBR5. Upon neural induction of HD Q71-iPSC line #2 with downregulated levels of UBR5, NPCs accumulate mutant HTT aggregates as assessed by filter trap experiments with anti-polyQ-expansion diseases marker antibody. The images are representative of three independent experiments.

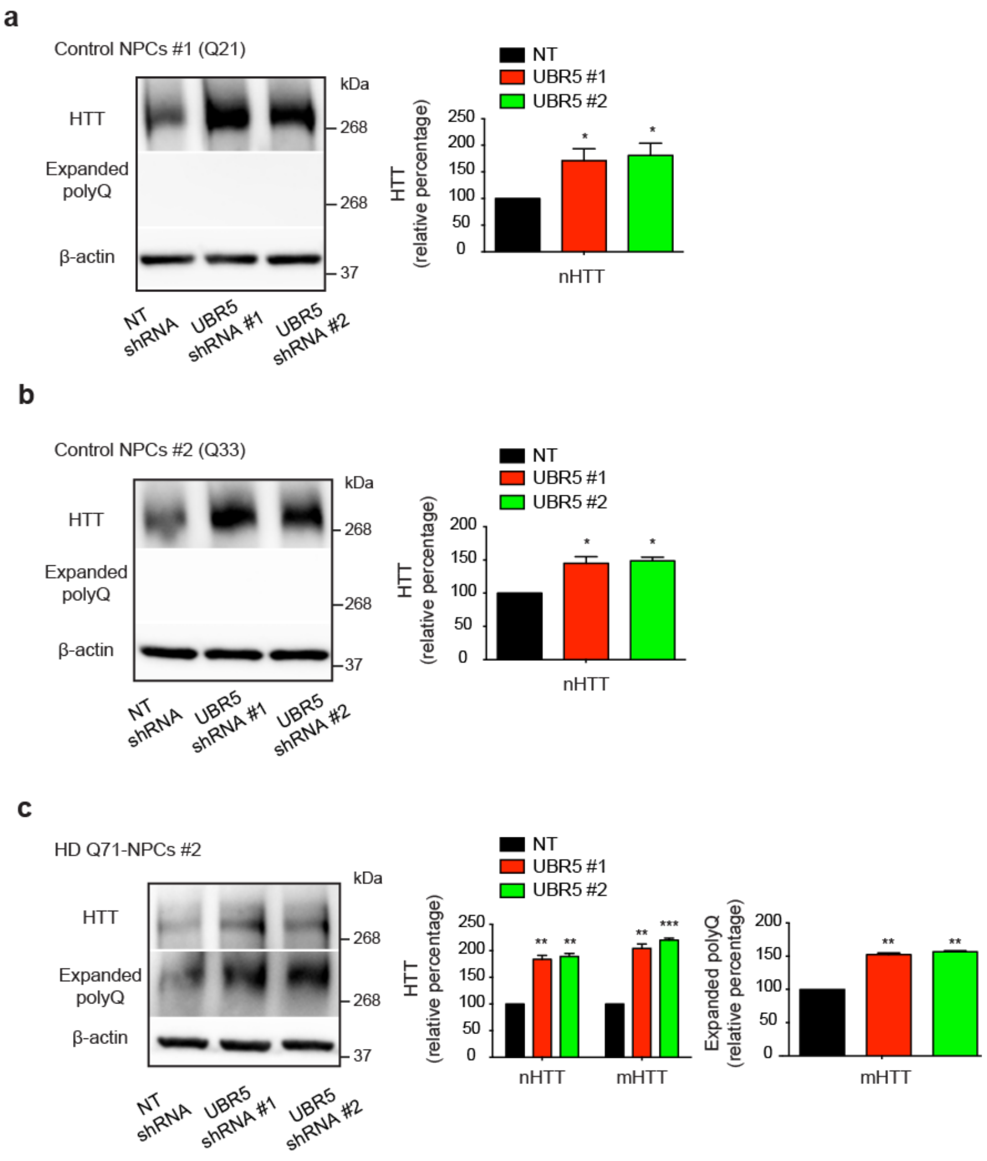

Supplementary Figure 29. iPSCs with downregulated levels of UBR5 differentiate into NPCs with high expression of HTT upon neural induction. a, Control iPSCs #1 with downregulated levels of UBR5 were differentiated into NPCs and analyzed by western blot. The graph represents the HTT relative percentage values to NT shRNA NPCs (corrected for β-actin) detected with antibody to total HTT (mean ± s.e.m. of three independent experiments). **b,** Western blot analysis of NPCs derived from control iPSCs #2 with downregulated levels of UBR5. The graph represents the HTT relative percentage values to NT shRNA NPCs (corrected for β-actin) detected with antibody to total HTT (mean ± s.e.m. of three independent experiments). **c,** Western blot analysis of NPCs derived from HD Q71-iPSCs #2 with downregulated levels of UBR5. The graph represents the HTT relative percentage values to NT shRNA NPCs (corrected for β-actin) detected with antibody to total HTT (mean ± s.e.m. of two independent experiments). All the statistical comparisons were made by Student's t-test for unpaired samples. P-value: *(P<0.05), **(P<0.01), ***(P<0.001).

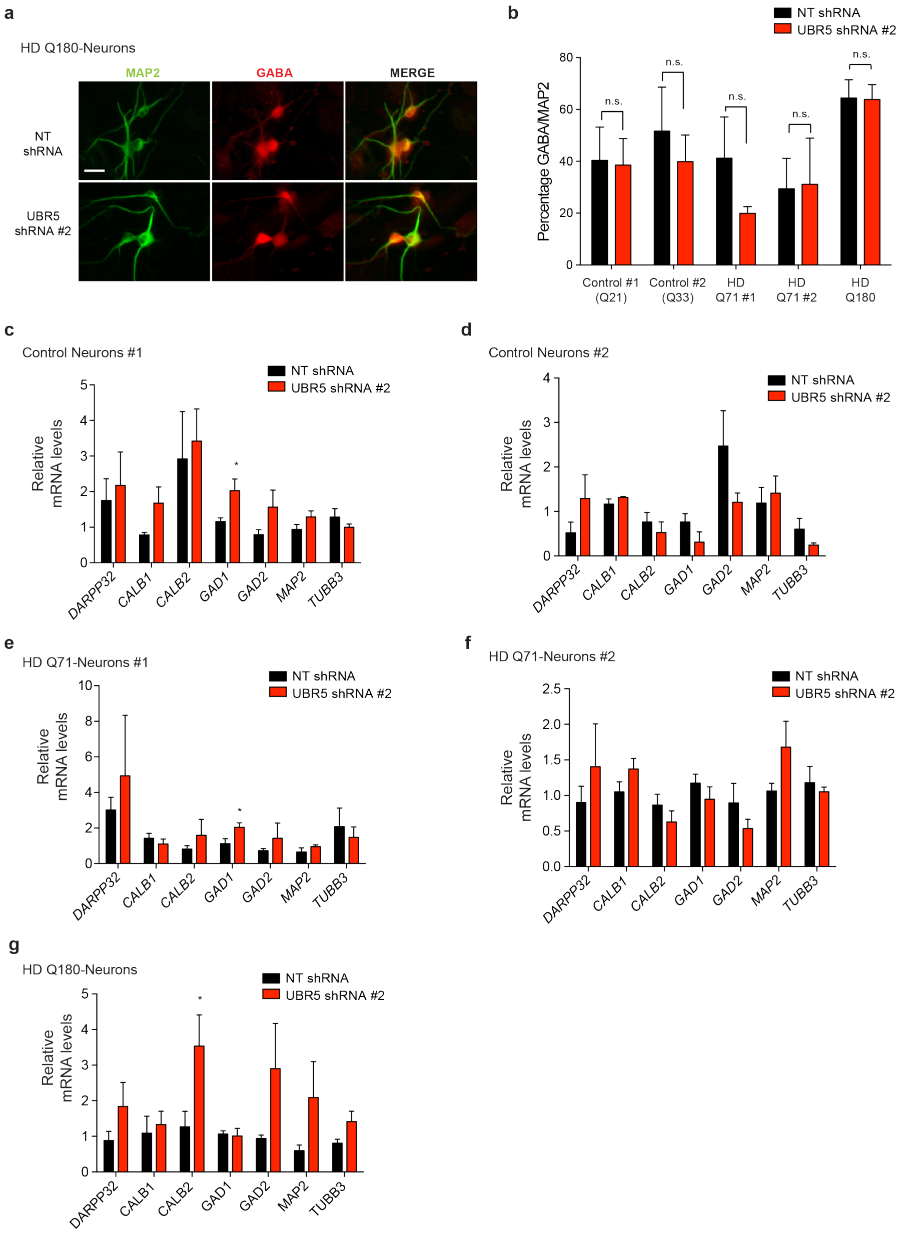

Supplementary Figure 30. iPSCs with downregulated levels of UBR5 can differentiate into striatal neurons. a, Striatal neuronal differentiation of HD Q180-iPSCs with downregulated levels of UBR5. GABA staining was used as a marker of striatal neurons. MAP2 and Hoechst 33342 staining were used as markers of neurons and nuclei, respectively. Scale bar represents 20 µm. The images are representative of three independent experiments. **b,** Quantification of GABA cell populations among MAP2-positive neurons upon striatal neuronal differentiation (mean ± s.e.m., three independent experiments, 1500-2000 cells per condition for each line). Knockdown of UBR5 was performed at the iPSC stage. **c,** qPCR analysis of striatal neuronal markers (*DARPP32*, *CALB1*, *CALB2*, *GAD1*, *GAD2*) and pan-neuronal markers (*MAP2*, *TUBB3*) in neurons derived from control iPSCs #1 with downregulated levels of UBR5. The graph represents the relative expression to NT shRNA neurons (mean ± s.e.m. (n= 4 independent experiments)). **d,** Control neurons #2 (n= 3 independent experiments). **e,** HD Q71-Neurons #1 (n= 3 independent experiments). **f,** HD Q71-Neurons #2 (n= 2 independent experiments). **g,** HD Q180-Neurons (n= 3 independent experiments). All the statistical comparisons were made by Student's t-test for unpaired samples. P-value: *(P<0.05).

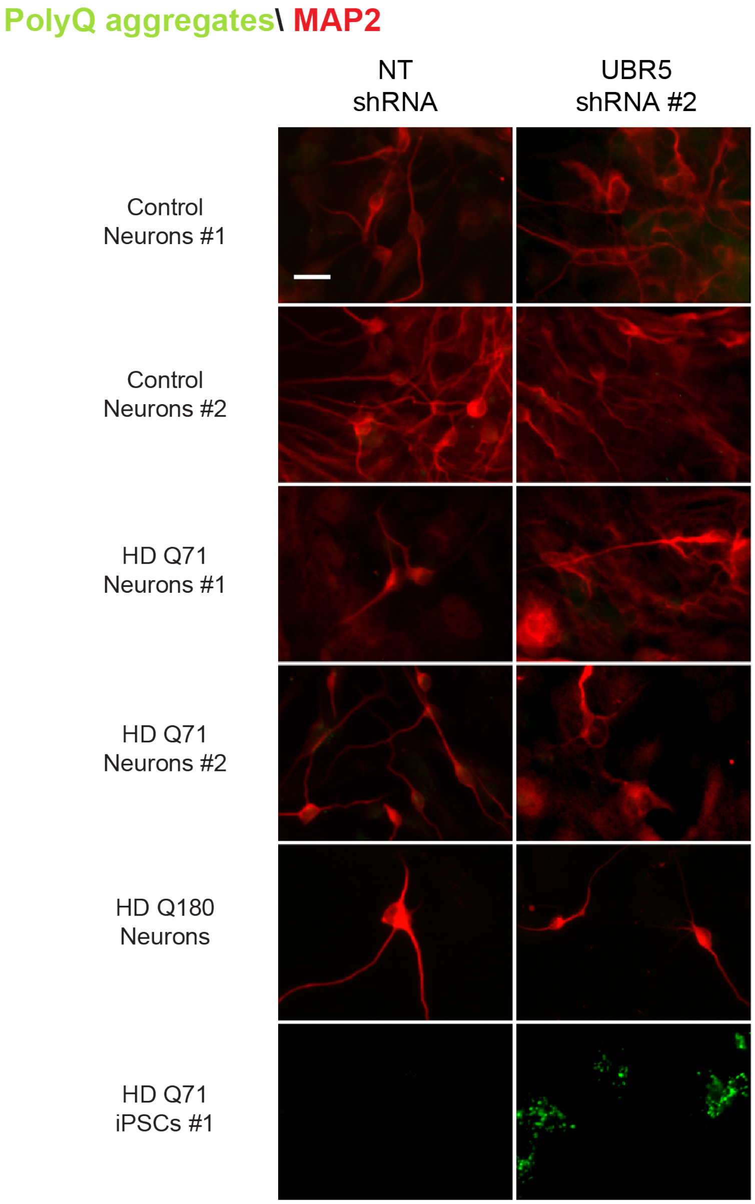

Supplementary Figure 31. Lack of polyQ-expanded HTT aggregates in terminally differentiated neurons derived from HD-iPSCs with downregulated levels of UBR5. Striatal neuronal differentiation of distinct control and HD-iPSCs with downregulated levels of UBR5. PolyQ-expanded and MAP2 staining were used as markers of polyQ-expanded aggregates and neurons, respectively. Scale bar represents 20 µm. The images are representative of three independent experiments.

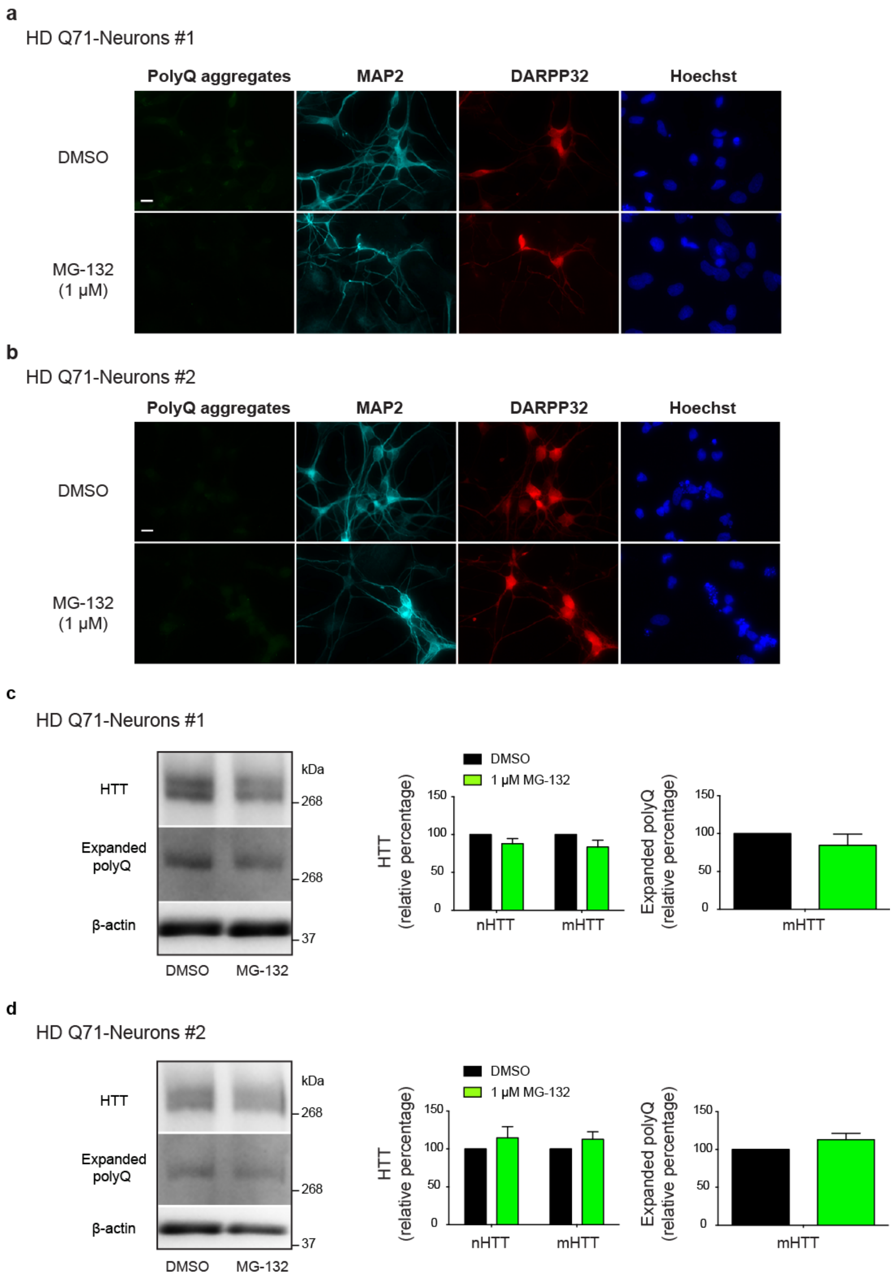

Supplementary Figure 32. Proteasome inhibition does not trigger aggregation of polyQ-expanded HTT in MSNs derived from HD-iPSCs. a, b, Immunocytochemistry with antibody against polyQ-expanded proteins of HD Q71-neurons lines #1 (**a**) and #2 (**b**) treated with 1 µM MG-132 for 16 h. MAP2 was used as a pan-neuronal marker whereas DARPP32 was used as a marker of striatal neurons. Cell nuclei were stained with Hoechst 33342. Scale bar represents 10 µm. The images are representative of two independent experiments. **c, d,** Western blot analysis of HD Q71-neurons lines #1 (**c**) and #2 (**d**) treated with 1 µM MG-132 for 16 h. The graphs represent the relative percentage values to the respective DMSO-treated neurons (corrected for β-actin) of normal (nHTT) and mutant HTT (mHTT) detected with antibodies to total HTT and polyQ-expanded proteins (mean ± s.e.m. of two independent experiments). No significant differences were found. All the statistical comparisons were made by Student's t-test for unpaired samples.

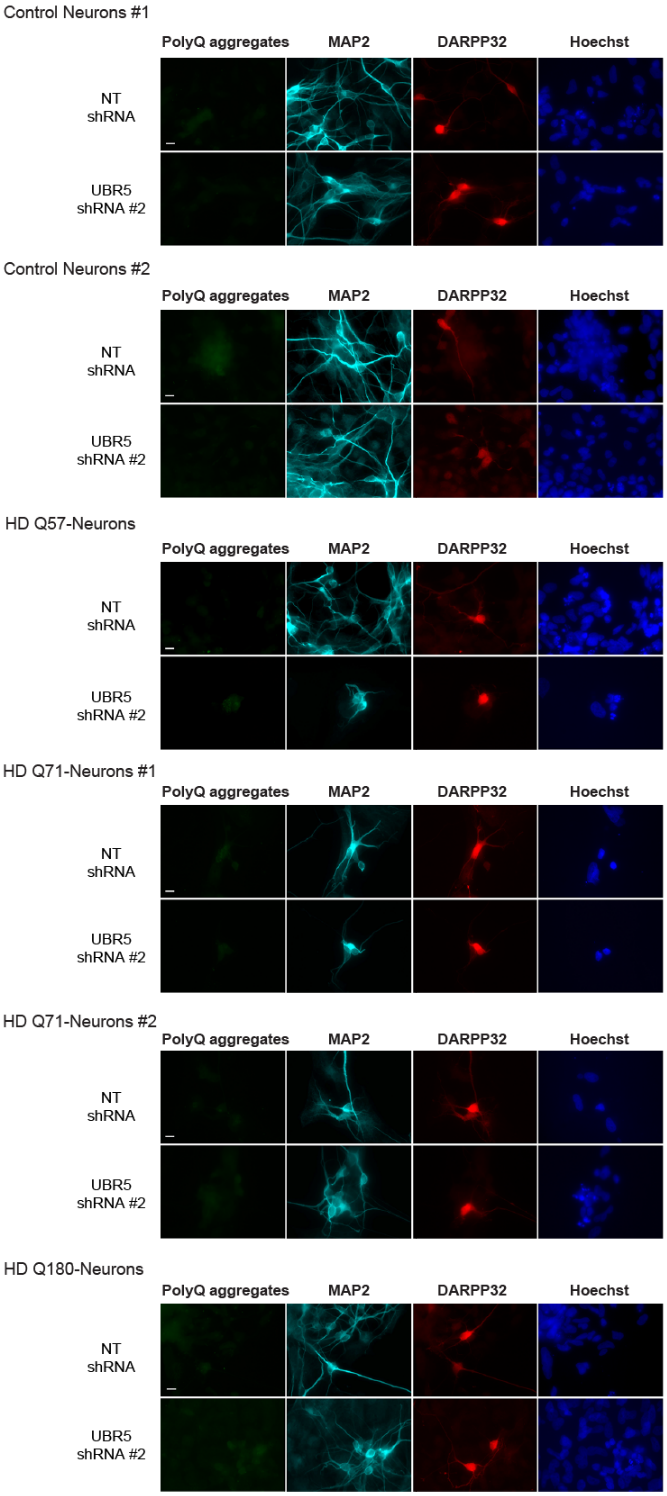

Supplementary Figure 33. Loss of UBR5 does not induce aggregation of mutant HTT in MSNs derived from HD-iPSCs. Immunocytochemistry with antibody against polyQ-expanded proteins in the indicated neurons. MAP2 was used as a pan-neuronal marker whereas DARPP32 was used as a marker of striatal neurons. Cell nuclei were stained with Hoechst 33342. Scale bar represents 10 µm. The images are representative of three independent experiments.

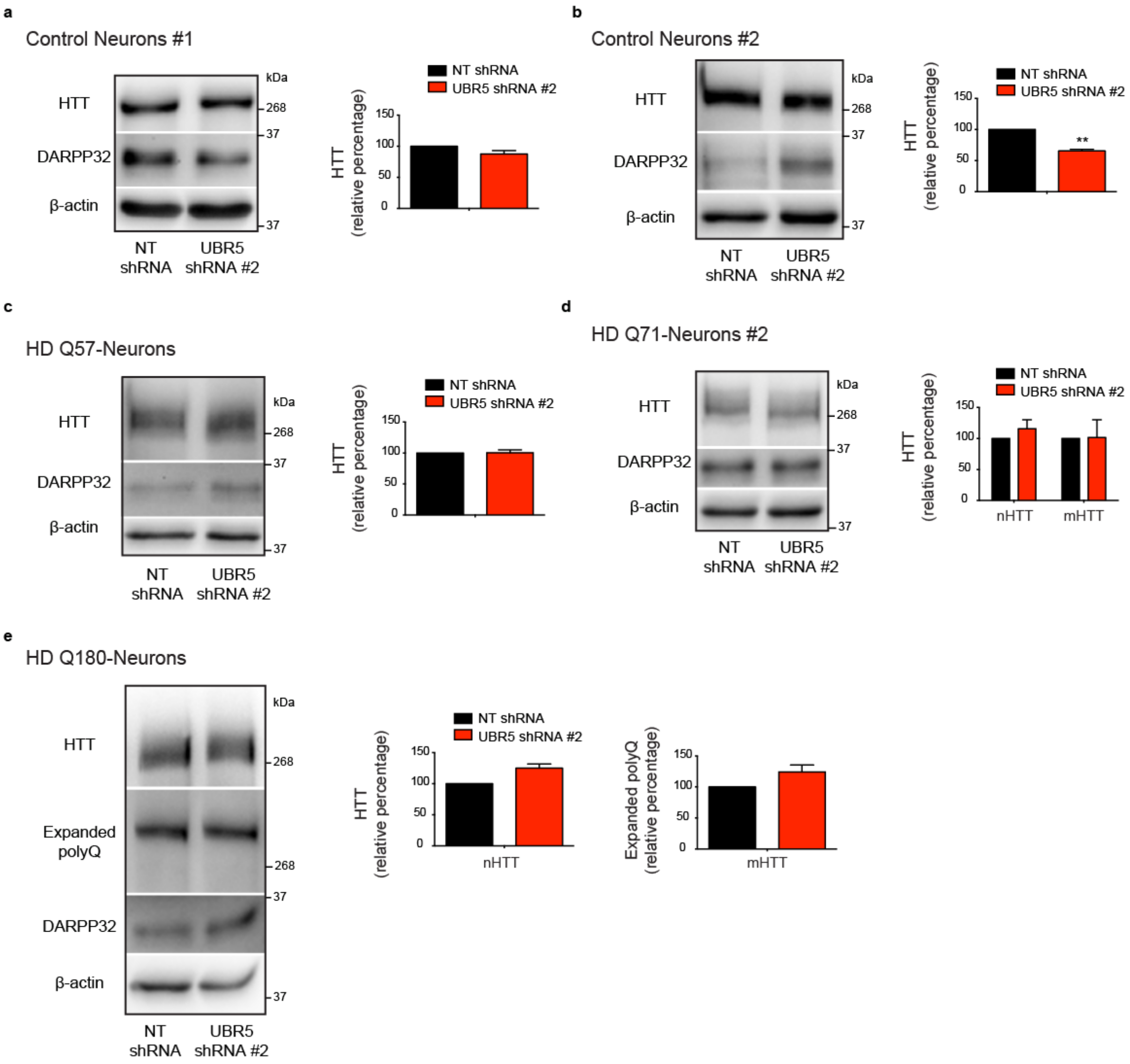

Supplementary Figure 34. Knockdown of UBR5 in MSN cultures derived from iPSCs does not increase HTT levels. a, b, Western blot analysis of the indicated control neuronal cultures with antibodies to HTT and DARPP32. The graphs represent the HTT relative percentage values to the respective NT shRNA neurons corrected for β-actin loading control (mean ± s.e.m. of three independent experiments). **c,** Western blot analysis of HD Q57-neurons. The graph represents the relative percentage value to NT shRNA neurons (corrected for β-actin) of total HTT levels detected with anti-HTT antibody (mean ± s.e.m. of three independent experiments). **d,** Western blot analysis of HD Q71-neurons #2. The graph represents the relative percentage values to NT shRNA neurons (corrected for β-actin) of nHTT and mHTT detected with anti-HTT antibody (mean ± s.e.m. of four independent experiments). **e,** Western blot analysis of HD Q180-neurons. The graphs represent the relative percentage values to NT shRNA neurons (corrected for β-actin) of nHTT and mHTT detected with antibodies to HTT and polyQ-expanded proteins, respectively (mean ± s.e.m. of three independent experiments).

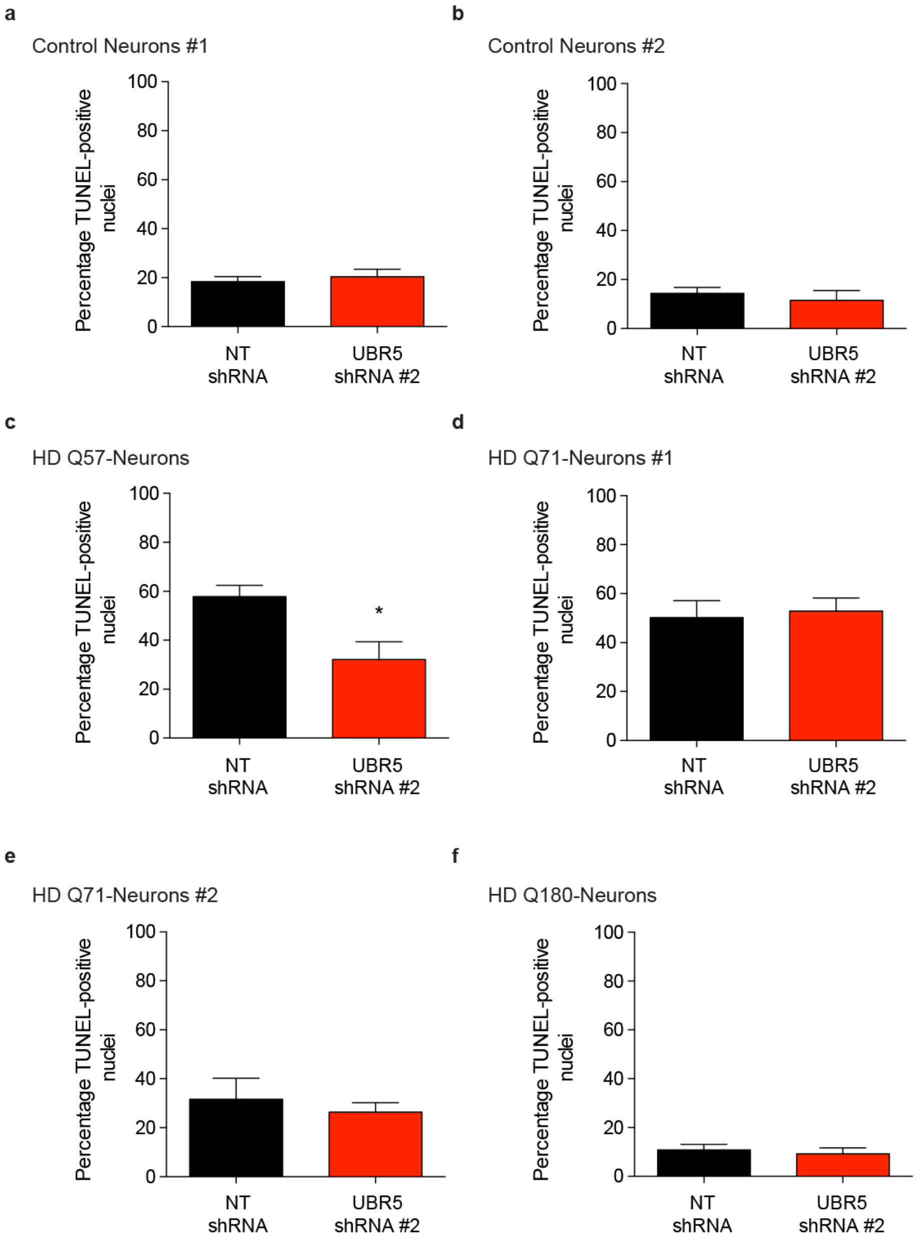

Supplementary Figure 35. Loss of UBR5 does not induce apoptosis in MSN cultures derived from iPSCs. Percentage of TUNEL-positive nuclei in the indicated MSN cultures: **a,** Control Neurons #1, **b,** Control Neurons #2, **c,** HD Q57-Neurons, **d,** HD Q71-Neurons #1, **e,** HD Q71-Neurons #2, **f,** HD Q180-Neurons. In **a-f**, total number of cells was evidenced after staining of nuclei with Hoechst 33342. Each graph represents the mean ± s.e.m. of the percentage observed in 3 independent neuronal differentiation experiments (we assessed approximately 200-300 total nuclei in each independent experiment for each line). All the statistical comparisons were made by Student's t-test for unpaired samples. P-value: *(P<0.05).

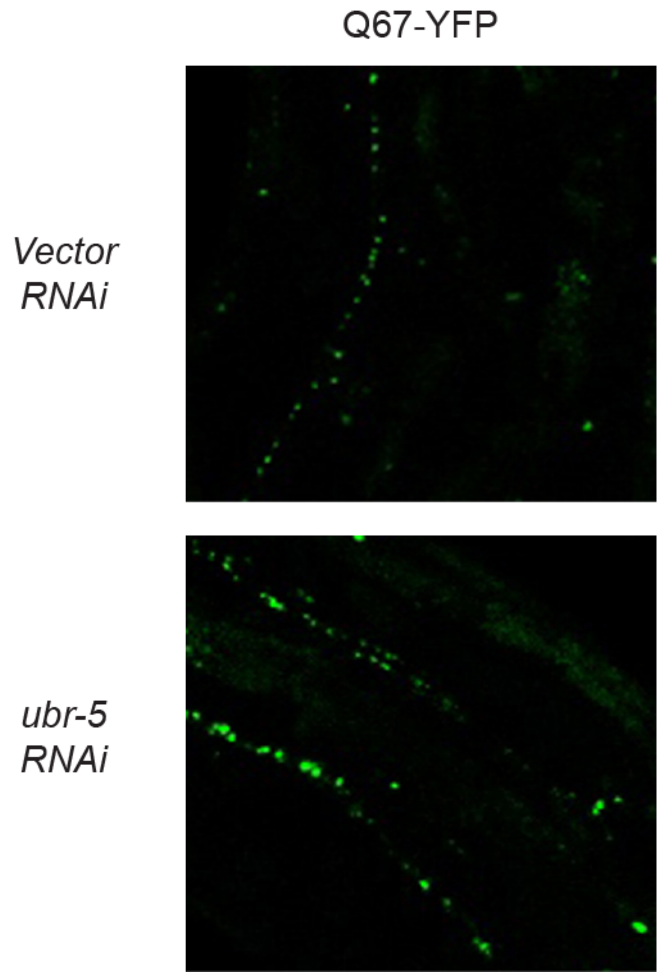

Supplementary Figure 36. Higher magnification of *C. elegans* mid-body of the images presented in **Figure 9c**.

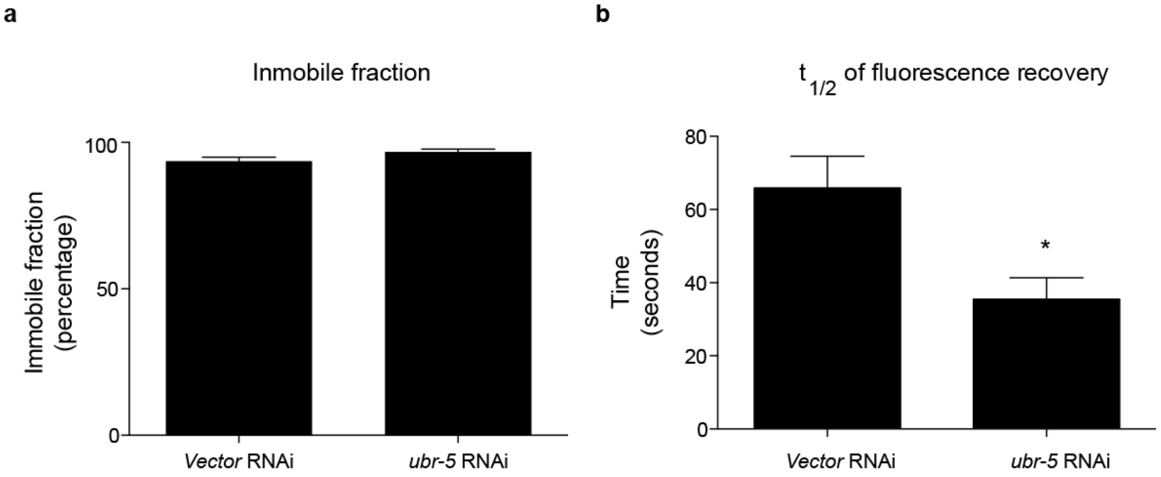

Supplementary Figure 37. FRAP analysis of Q67-YFP-expressing worms. Worms fed with empty vector or *ubr-5* RNAi were immobilized and regions containing polyQ-YFP foci in the head neurons were bleached. Subsequently, pictures were taken once every 2 seconds for 90 times. **a,** Graph represents percentage of immobile fraction. **b,** Graph represents the half-life of fluorescence recovery. In (**a, b**) data represents the mean ± s.e.m. of 9 worms from two independent experiments. All the statistical comparisons were made by Student's t-test for unpaired samples. P-value: *(P<0.05).

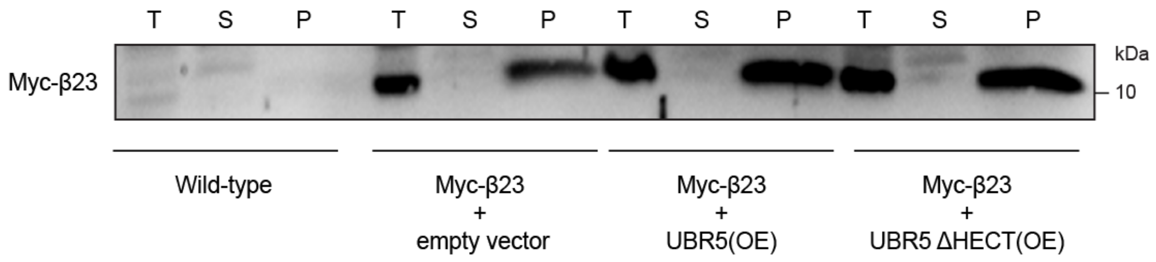

Supplementary Figure 38. Ectopic expression of UBR5 does not reduce aggregation of ß-amyloid fibrils. Upon cell fractionation of HEK293 cells, ß23 polypeptide with N-terminal c-Myc-epitope is largely recovered in the insoluble fraction. Overexpression of UBR5 does not increase the solubility of ß23 fibrils, as we did not detect the protein in the soluble fraction under this condition. Anti-Myc antibody was used to detect ß23 protein in the distinct cell fractions. T, total lysate; S, soluble fraction; P, pellet fraction. The images are representative of four independent experiments.

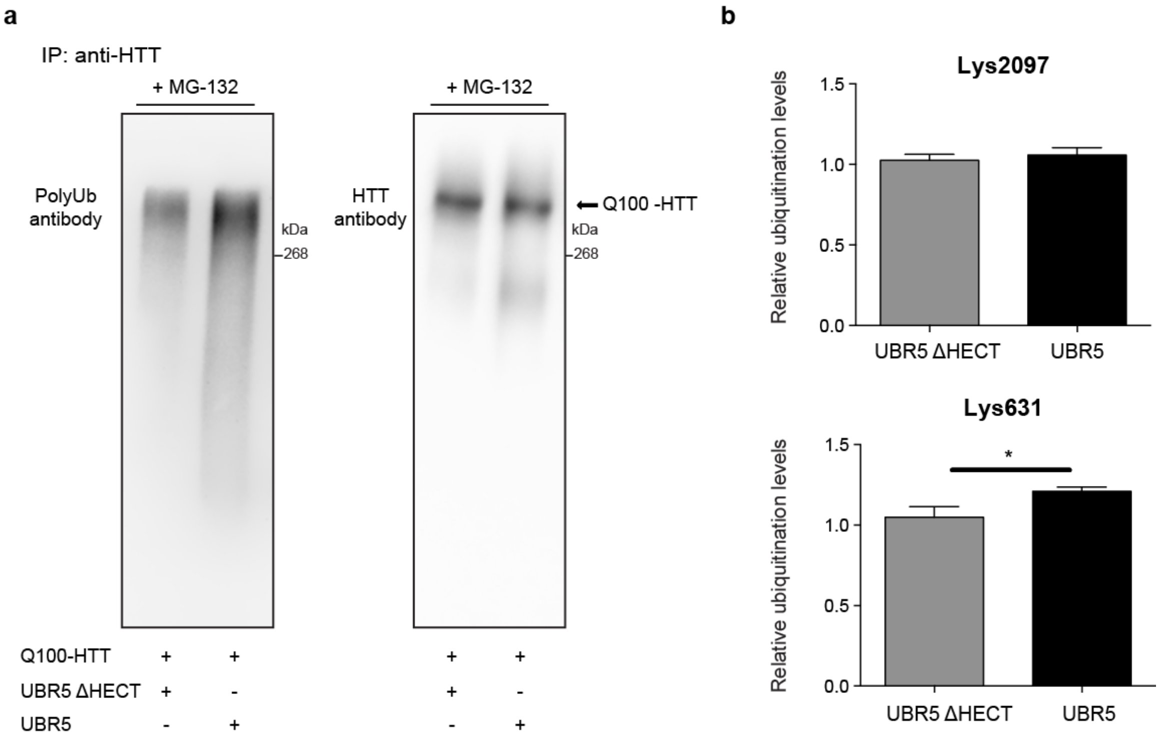

Supplementary Figure 39. Identification of HTT ubiquitination sites. **a,** Immunoprecipitation with anti-HTT antibody in Q100-HTT(OE) HEK293 cells treated with 0.5 µM MG-132 for 16 h. Immunoprecipitation was followed by western blot with antibodies to HTT and polyubiquitinated proteins (polyUb) to detect immunoprecipitated total HTT protein and polyUb-HTT, respectively. These data validate increased polyubiquitination of HTT upon wild-type UBR5 overexpression when compared to catalytic inactive UBR5 mutant of the samples analyzed by proteomics for identification of ubiquitination sites. **b,** Relative ubiquitination levels of Lys2097 and Lys631 of HTT in Q100-HTT(OE) HEK293 cells upon overexpression of catalytic inactive mutant or wild-type UBR5. HTT was immunoprecipitated with anti-HTT antibody and analyzed by mass spectrometry. Lys631 and 2097 of HTT were found to be ubiquitinated. The ubiquitination intensity of each lysine was normalized by the total HTT level in every condition. The graphs show ubiquitination levels normalized to the levels of ubiquitination in samples overexpressing UBR5 catalytically catalytic inactive UBR5 mutant (mean ± s.e.m. of three independent experiments). All the statistical comparisons were made by Student's t-test for unpaired samples. P-value: *(P<0.05).

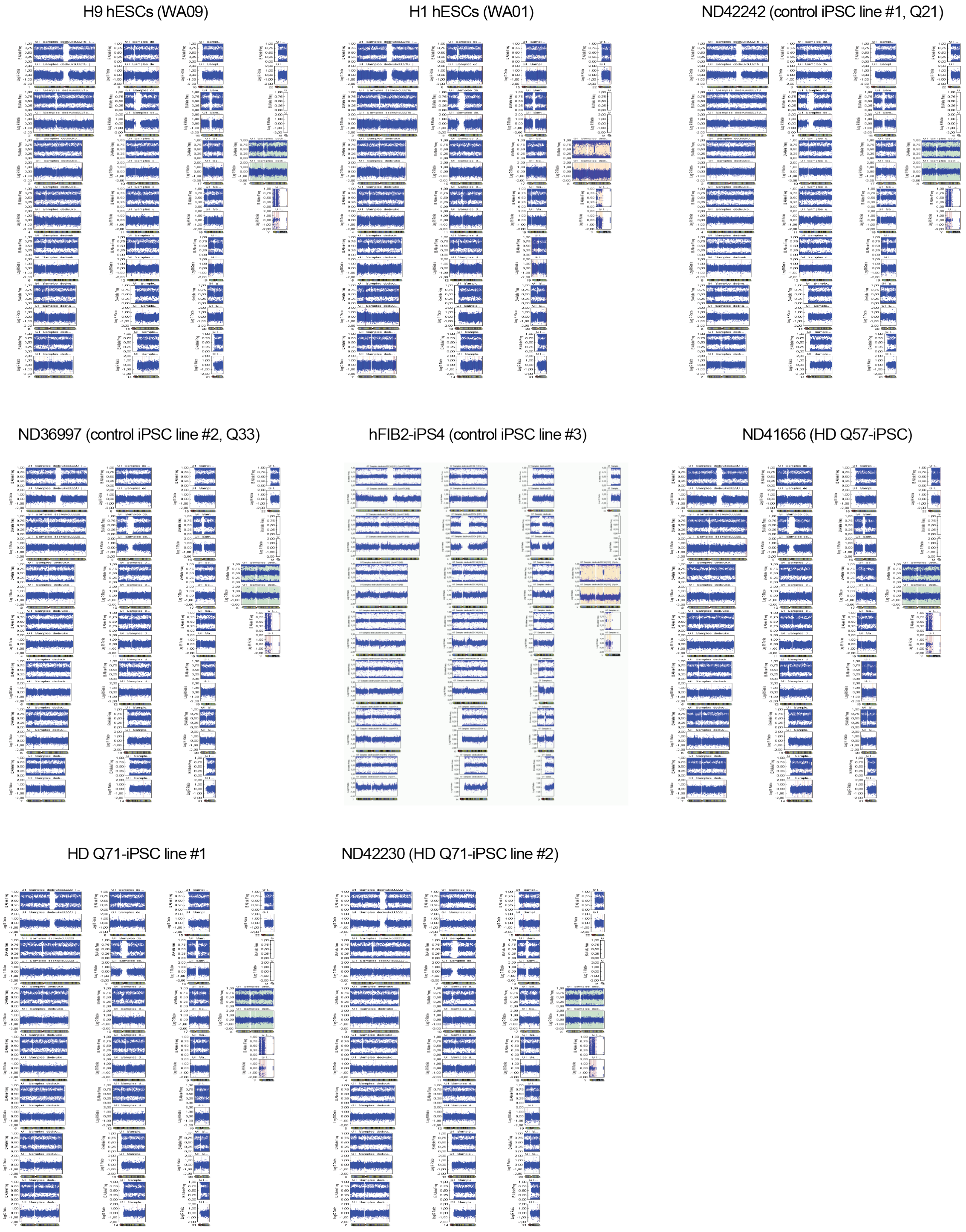

Supplementary Figure 40. SNP genotyping of hESCs and iPSCs used in this study.

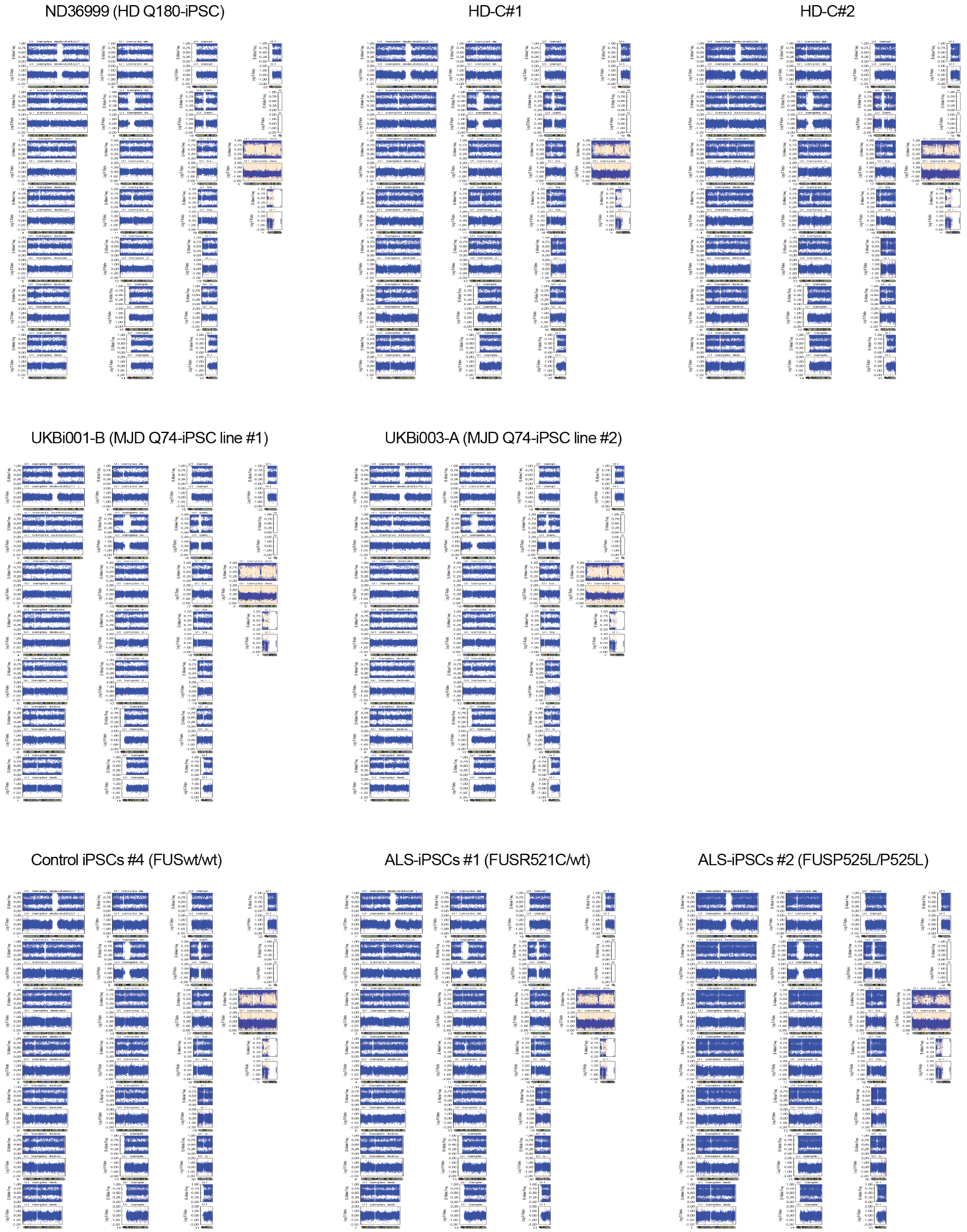

Supplementary Figure 40 (continuation). SNP genotyping of hESCs and iPSCs used in this study.

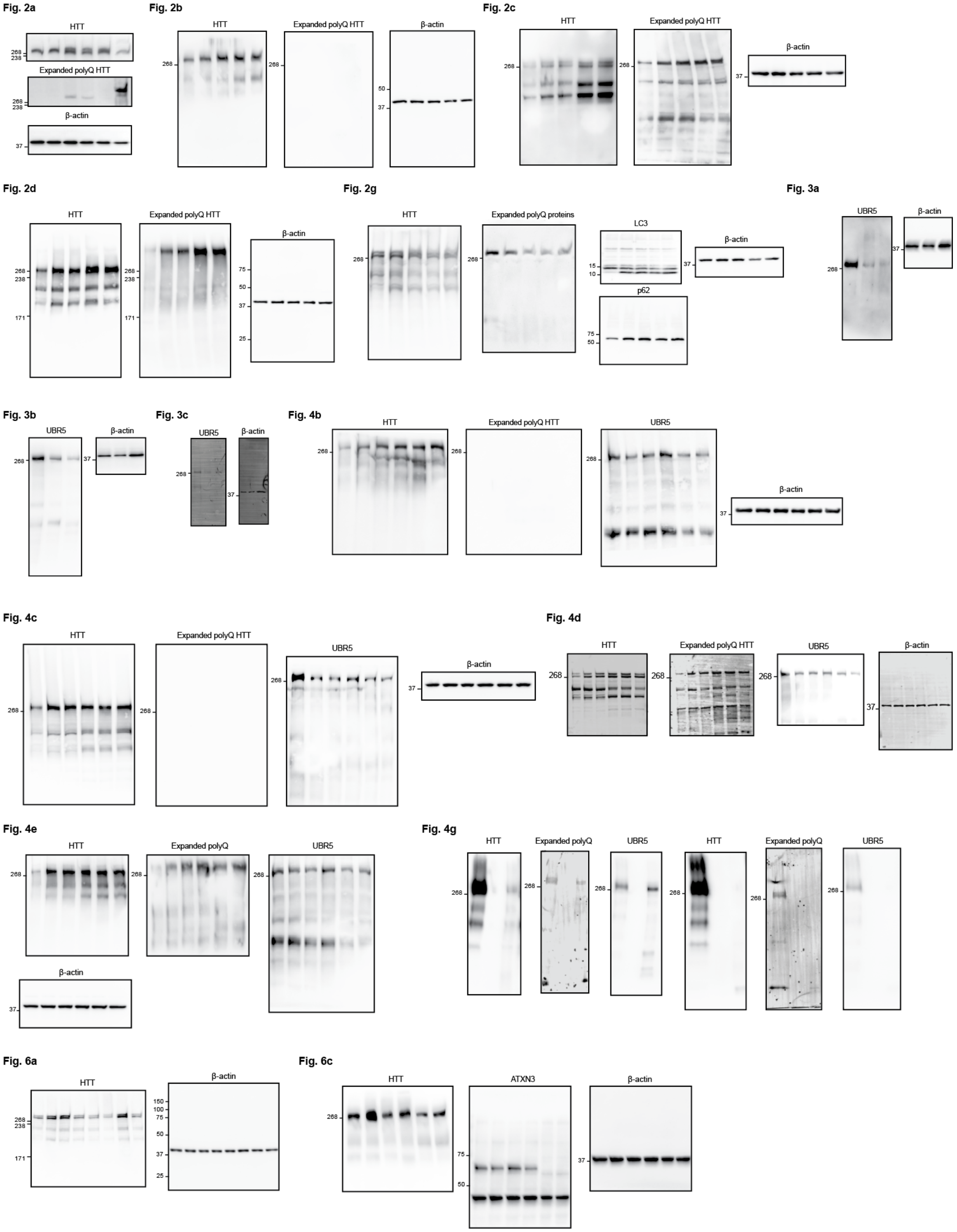

Supplementary Figure 41. Uncropped images are presented with molecular weight ladders.

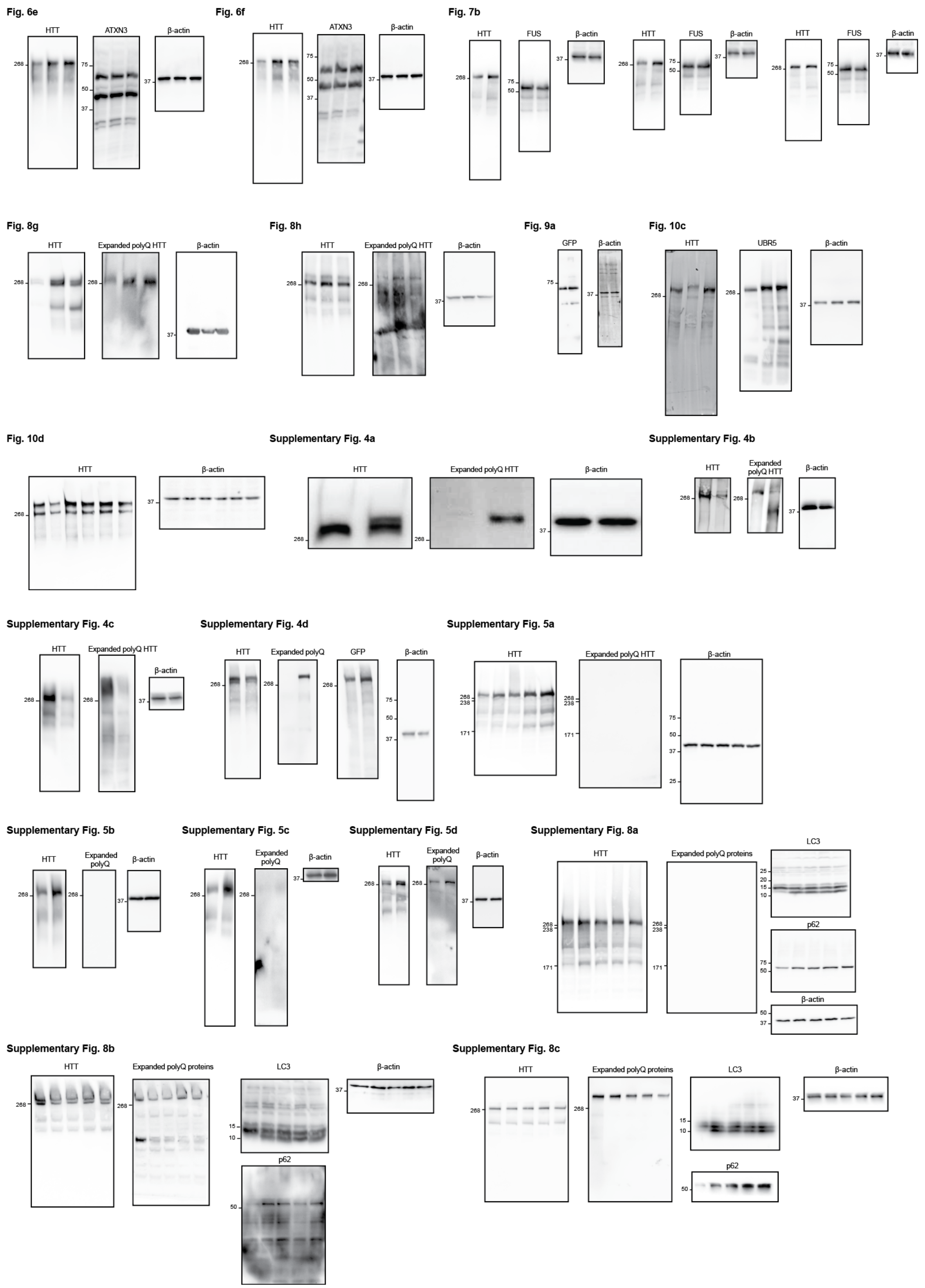

Supplementary Figure 41 (continuation). Uncropped images are presented with molecular weight ladders.

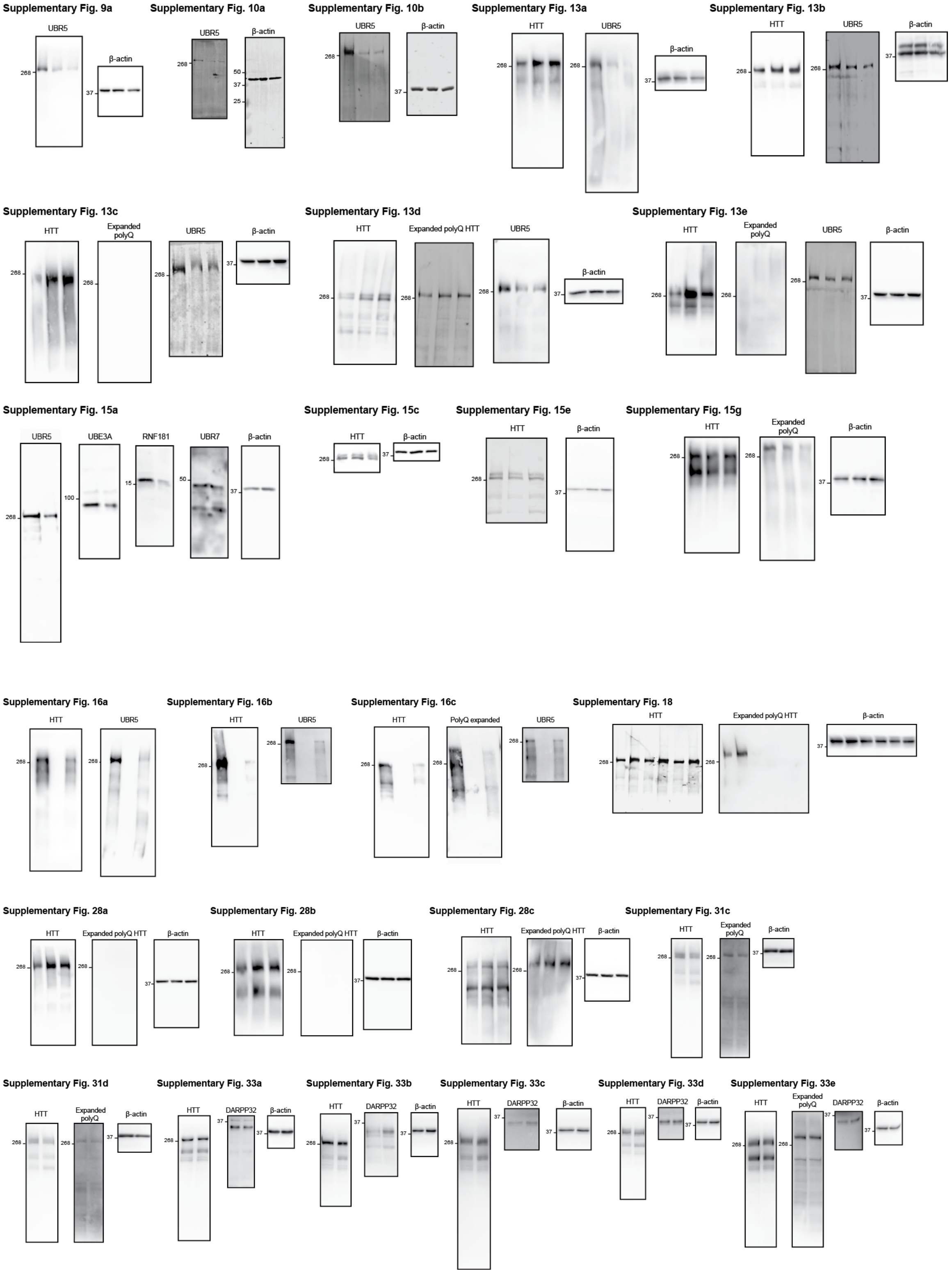

Supplementary Figure 41 (continuation). Uncropped images are presented with molecular weight ladders.

iPSC line	Age at sampling	Age of onset	Sex	Relevant gene for disease	Clinical information
**HD Q71 lines #1/#2**	20	14	Female	Q71HTT/ Q19HTT	Rigid form of HD, hypokinetic variant of HD with dystonia and marked tremor, ataxic wide-based gait, symptomatic
**HD Q180**	6	<6	Male	Q180HTT/ Q18HTT	Juvenile HD, symptomatic
**HD Q57**	29		Female	Q57HTT/ Q17HTT	Post-juvenile HD, pre-symptomatic
**MJD Q74 line #1**	40	30	Male	Q74ATXN3/ Q21ATXN3	Scale for the Assessment and Rating of Ataxia (SARA): 23,0. Additional symptoms: parkinso nism, restless legs, spasticity, neuropathy
**MJD Q74 line #2**	38	31	Male	Q74ATXN3/ Q22ATXN3	SARA: 22,5. Additional symptoms: parkinso nism, restless legs, spasticity, neuropathy
**ALS line #1**	39	Mid-late age	Male	$FUS^{R521C/WT}$	Affected by ALS in mid-late age
**ALS line #2**	Generated from control iPSCs #4 by TALEN-directed mutagenesis		Male	$FUS^{P525L/P525L}$	Patient carrying this mutation displayed severe and juvenile onset of ALS

Supplementary Table 1. Patient information for disease-iPSC lines.

E3 ubiquitin ligases increased in hESCs	hESC vs neuron ratio	q-value
**TRIM71**	17.26	0.029
**TRIM28**	2.61	0.015
**UBR5**	2.49	0.007
**RGPD1**	2.46	0.010
**RGPD2**	2.41	0.010
**RGPD8**	2.36	0.013
**UBR7**	2.35	0.007
**RGPD4**	2.26	0.011
**SART1**	2.26	0.011
**RGPD5**	2.26	0.011
**TRIM33**	2.24	0.009
**RGPD3**	2.19	0.012
**UBE4B**	1.95	0.011
**RNF20**	1.94	0.017
**ARIH2**	1.80	0.037
**RNF181**	1.69	0.004
**UBR4**	1.61	0.021
**PRPF19**	1.48	0.019
**HECTD1**	1.46	0.010
**KCMF1**	1.43	0.012
**UBE3A**	1.30	0.007
**TRIP12**	1.30	0.014
**RNF40**	1.27	0.034
**TRAF2**	1.21	0.008
**HERC1**	1.17	0.011
**HUWE1**	1.09	0.028

Supplementary Table 2. List of E3 enzymes significantly increased in hESCs compared with their neuronal counterparts. Tandem mass tag (TMT) quantitative proteomics comparing H9 hESCs with neurons. Statistical comparisons were made by Student's t-test (n= 3). False Discovery Rate (FDR) adjusted p-value (q-value) <0.05 was considered significant.

a)

Locus ID	Alleles	
	H9 hESCs	H1 hESCs
vWA	17, 17	15, 17
TH01	9.3, 9.3	9.3, 9.3
TPOX	10, 11	8,11
CSF1P0	11, 11	12, 13
D5S818	11, 12	9, 11
D13S317	9, 9	8, 11
D7S820	9, 11	8, 12
D16S539	12, 13	9,13

b)

Locus ID	Alleles	
	MJD-iPSC line #1	MJD iPSC line #2
TH01	6, 10	9, 9.3
CSF1P0	10, 11	9, 10
D5S818	12, 13	10, 12
D13S317	8, 10	11, 11
D7S820	10, 12	9, 11
D16S539	11, 14	9, 12

Supplementary Table 3. STR profiles of hESC and MJD-iPSC lines. a, The H9 and H1 hESCs used in our study matches exactly the published STR profile of these cells across the 8 STR loci analysed. **b,** The MJD-iPSCs used in our study matches exactly the STR profile of their parental fibroblasts provided by the depositor of the lines. The STR profile is not presented in full to protect the donor's identity as requested by the provider of the cells (EBiSC consortium).

	Allele size in base pairs					
	**TPOX**	**CSFIPO**	**THO1**	**vWA**	**D17S1303**	**D16S539**
	Allele1, Allele2	Allele1, Allele2	Allele1, Allele2		Allele1, Allele2	Allele1, Allele2
**ND30014**	242, 242	306, 318	169, 172	146, 151	223, 227	154, 159
**Control iPSCs #1**	242, 242	306, 318	169, 172	146, 151	223, 227	154, 159
**GMO2183**	231, 231	315, 315	169, 172	146, 159	231, 235	150, 154
**Control iPSCs #2**	231, 231	315, 315	169, 172	146, 159	231, 235	150, 154
**GMO4281**	231, 242	306, 310	165, 169	146, 154	223, 223	154, 159
**HD Q71-iPSCs #1**	231, 242	306, 310	165, 169	146, 154	223, 223	154, 159
**HD Q71-iPSCs #2**	231, 242	306, 310	165, 169	146, 154	223, 223	154, 159
**GMO9197**	231, 234	306, 310	155, 172	135, 145	231, 235	142, 159
**HD Q180-iPSCs**	231, 234	306, 310	155, 172	135, 145	231, 235	142, 159
**HD-C#1 (isogenic corrected line)**	231, 234	306, 310	155, 172	135, 145	231, 235	142, 159
**HD-C#2 (isogenic corrected line)**	231, 234	306, 310	155, 172	135, 145	231, 235	142, 159
**ND33392**	237, 241	309, 314	164, 171	140, 145	223, 235	154, 159
**HD Q57-iPSCs**	237, 241	309, 314	164, 171	140, 145	223, 235	154, 159
**Control iPSCs #4**	231, 231	306, 311	169, 172	140, 155	223, 235	159, 168
**ALS-iPSCs #2**	231, 231	306, 311	169, 172	140, 155	223, 235	159, 168

Supplementary Table 4. Confirmation of genetic identity of HD-iPSCs lines with the corresponding parental fibroblasts by STR analysis. Since ALS-iPSCs #2 were raised from control iPSCs #4 by TALEN-directed mutagenesis, we confirmed that the STR profile of the ALS-iPSCs #2 used in our experiments matches with the profile of control iPSCs #4.

Gene	Forward (5' → 3')	Reverse (5' → 3')
ACTB	CTGGCACCCAGCACAATG	CCGATCCACACGGAGTACTTG
GAPDH	GCACCGTCAAGGCTGAGAAC	GGATCTCGCTCCTGGAAGATG
UBR5	TGGAGTGAATCTGAGCCTTACAGA	AATGTTGCTCGTGGATGATGTAAT
HTT	GATTGGATGGGCACCATTAGA	GATTGGATGGGCACCATTAGA
0CT4	GGAGGAAGCTGACAACAATGAAA	GGCCTGCACGAGGGTTT
NANOG	AAATCTAAGAGGTGGCAGAAAAACA	GCCTTCTGCGTCACACCATT
SOX2	TGCGAGCGCTGCACAT	TCATGAGCGTCTTGGTTTTCC
DPPA4	CTGGTGCCAACAATTGAAGCT	AGGCACACAGGCGCTTATATG
GATA6	AGCGCGTGCCTTCATCA	GTGGTAGTTGTGGTGTGACAGTTG
AFP	GAGGGAGCGGCTGACATTATT	ACCAGGGTTTACTGGAGTCATTTC
MSX1	CTCCGCAAACACAAGACGAAC	CACATGGGCCGTGTAGAGTC
NES	TGAAGGGCAATCACAACAGG	TGACCCCAACATGACCTCTG
PAX6	CATACCAAGCGTGTCATCAATAAAC	TGCGCCCATCTGTTGCT
UBE3A	GACTCAAAGTTAGACGTGACCATATCA	CTTCAAGTCTGCAGGATTTTCCA
UBR7	TGCCGGCTCTAGTTCTGAATC	TTCTGCGTTGAGGCTTTCATT
RNF181	CCTTGCCATCACCTTTTCCA	GGACAGGAATTTGTCTTGCTTAG
TRIM71	CGGCGAGGCATAAGAAAGC	GCTTTCACCTGGCGGATCT
DARPP32	CCTGAAGGTCATCAGGCAGT	GGTCTTCCACTTGGTCCTCA
CALB1	TCAGGACGGCAATGGATACA	AAGAGCAAGATCCGTTCGGT
CALB2	TGGCGGAAGTACGACACAGA	GGAATCCCTTGAGCTCATTGG
GAD1	CCAAGGTGCTGGACTTTCAT	AAATCGAGGATGACCTGTGC
GAD2	CGAGCCTGGTGCCAAGTG	CAGGGCGCACAGTTTGTTT
MAP2	AAAGAAGCTCAACATAAAGACCAGACT	GTGGAGAAGGAGGCAGATTAGC
TUBB3	GGCCAAGTTCTGGGAAGTCA	CGAGTCGCCCACGTAGTTG

Supplementary Table 5. List of primers used for qPCR experiments.
